# Narrowband Emitting Tin Dion–Jacobson Perovskites through Systematic Spacer Cation Design to Suppress Metal Lone-Pair Anisotropy

**DOI:** 10.1021/jacs.6c11969

**Published:** 2026-10-01

**Authors:** Kristel M. Forlano, Jiahao Xie, Christopher T. Triggs, Jalianet Román-Matías, Daniel D. Kohler, Francesca Canestra, Cristina Femoni, David P. Lafayette, Ilia A. Guzei, Daniele Cortecchia, John C. Wright, Yanfa Yan, Song Jin

**Affiliations:** † Department of Chemistry, 5228 University of Wisconsin-Madison, Madison, Wisconsin 53706, United States; ‡ Wright Center for Photovoltaics Innovation and Commercialization, Department of Physics and Astronomy, University of Toledo, Toledo, Ohio 43606, United States; § Department of Industrial Chemistry Toso Montanari, 9296 University of Bologna, Bologna 40129, Italy

## Abstract

Two-dimensional (2D) Dion-Jacobson (DJ) tin perovskites are promising lead-free semiconductors, yet the limited number of phases reported and their frequently broad and asymmetric photoluminescence (PL) have hindered mechanistic understanding and rational design of their optoelectronic properties. Here, we systematically study 13 DJ Pb/Sn iodide perovskite pairs with identical diammonium spacer cations, including four newly synthesized Sn phases, to seek structure–property trends. We show that narrow and symmetric PL of Pb perovskites alone does not predict similarly favorable emission from Sn analogues. Instead, Sn DJ perovskites with narrow and symmetric PL share a structural motif defined by crystallographically equivalent perovskite pockets and minimal metal off-centering distortion, which can be achieved by symmetric spacer cations. First-principles calculations together with symbolic regression quantitatively connect this motif to reduced anisotropy of the Sn^2+^ lone pair expression and smaller excited-state structural reorganization, consistent with suppressed vibronic broadening of PL peaks. These structural and electronic criteria guide the identification and synthesis of (4F-PDMA)­SnI_4_ (4F-PDMA = 2,3,5,6-tetrafluoro-1,4-phenylenedimethanammonium) that exhibits anticipated structural features, favorable PL characteristics, and excellent stability. This work introduces a rational spacer cation design strategy to suppress metal lone-pair anisotropy and achieve tin DJ perovskites with narrow PL emission for future high-performance optoelectronic applications.

## Graphical abstract

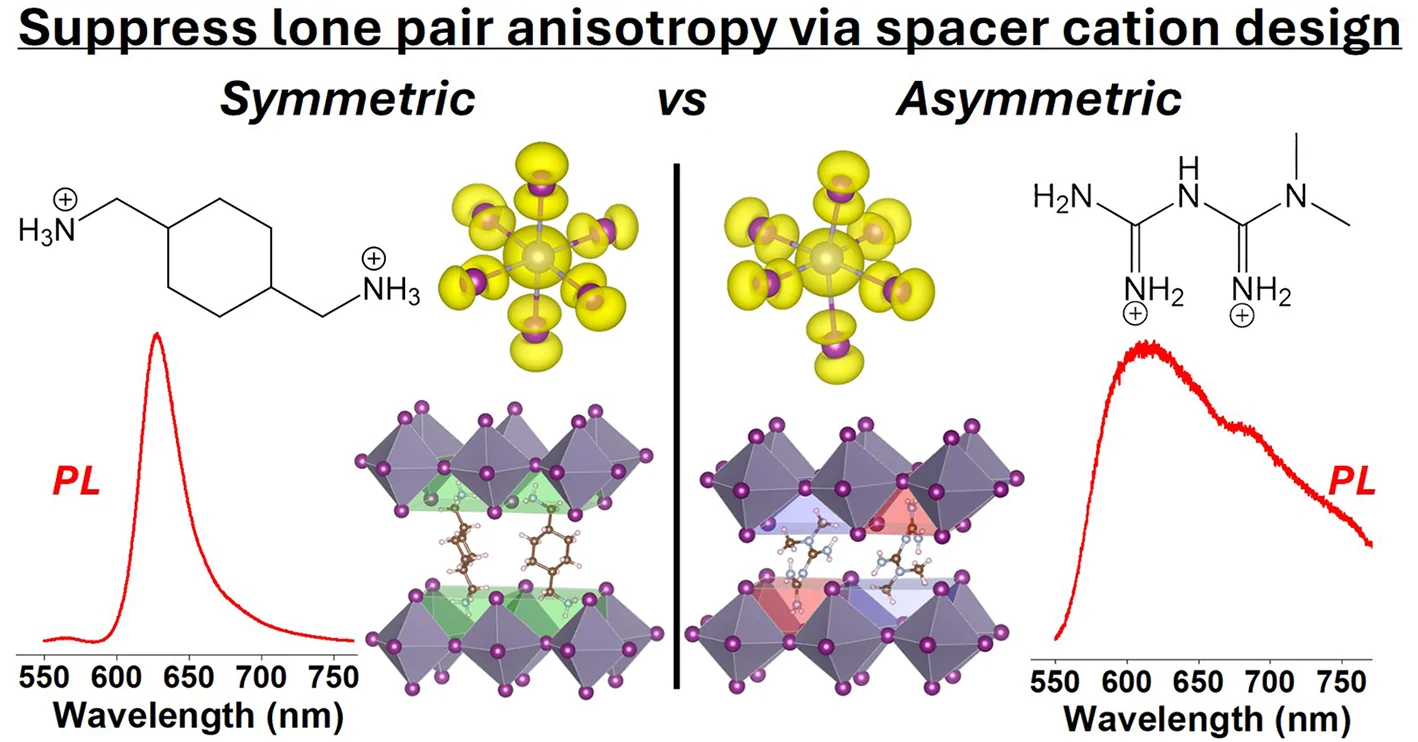

## Introduction

Two-dimensional (2D) metal halide perovskites are tunable semiconductor materials promising for high performance solar cells,^1–6^ light-emitting diodes,^7,8^ lasing,^9–12^ thin-film transistors,^13–16^ photodetectors,^17,18^ and other optoelectronic applications. The general formula of 2D perovskites is (LA)_*y*_(A)_*n*‑1_(B)_*n*_(X)_3*n*+1_,^19–21^ where B is a divalent metal (such as Pb^2+^, Sn^2+^, or Ge^2+^), X is a halide (such as I^–^, Br^–^, or Cl^–^), A is a small monovalent cation (such as methylammonium), and LA is a large organic spacer cation that bisects the inorganic networks made up of corner-sharing [BX_6_]^4–^ octahedra into 2D slabs with discrete octahedral layer thickness defined by *n*. The valency of different spacer cations leads to two primary 2D perovskite structure types: Ruddlesden–Popper (RP, *y* = 2) perovskites using monovalent spacer cations that create a bilayer of organic molecules held together by van der Waals forces and neighboring perovskite layers that are offset, and Dion-Jacobson (DJ, *y* = 1) perovskites using divalent spacer cations that form an interlayer without a van der Waals gap.^19–21^ The insulating organic layers with high dielectric contrast create a quantum well structure of the inorganic lattice, which greatly increases exciton binding energy and decreases excitonic lifetime.^22^ The compositional tunability of the 2D perovskite crystal structures allows for targeted design for specific optoelectronic properties.

Although RP perovskites dominate the 2D perovskite research, DJ perovskites are attractive due to their greater out-of-plane charge transport,^23–27^ higher resistance against ion diffusion,^28^ and improved air stability^6,29^ enabled by their inherently smaller interlayer spacing. Moreover, the increased structural rigidity imposed by the diammonium spacer cation monolayer has been associated with reduced electron–phonon coupling.^23,30–32^ These qualities have led to the successful utilization of DJ lead iodide perovskites in both 2D perovskite and 2D/3D perovskite heterojunction solar cells.^3,6,33–36^ These successes motivated a number of exploratory and systematic experimental, theoretical, and/or data-driven studies on the structural chemistry and photophysical properties of DJ lead iodide perovskites across diverse types of diammonium spacer cations (aliphatic, cyclic, aromatic, symmetric or asymmetric) and how their formation and stability could depend on the structural features of the spacer cations.^36–44^

Beyond these studies on lead-based DJ perovskites, lead-free tin-based DJ perovskites are even less explored — prior to this study, fewer than 15 crystal structures of DJ tin iodide perovskites were reported in the Cambridge Crystallography Data Centre (CCDC) database. In addition to avoiding Pb toxicity, 2D Sn perovskites can exhibit attractive photophysical properties relative to Pb counterparts, including narrower bandgaps, lower exciton binding energies, high quantum efficiency, and the ability to lase.^9–11,45,46^ However, the poor air stability of Sn perovskites has historically limited their systematic studies and applications. The recent demonstration that DJ-forming spacer cations could improve the air stability of Sn perovskites^29^ motivates further study of the less explored Sn DJ perovskites. However, the very few reported Sn DJ perovskites often exhibit broad and dim photoluminescence (PL) with rather asymmetric peak shape,^14,37^ raising two central questions: whether the more extensive literature on Pb DJ perovskites can guide the discovery of Sn DJ perovskite analogues, and which spacer-controlled structural descriptors can determine or “predict” narrow and symmetric PL line shapes in Sn-based DJ perovskites. Such PL characteristics often correlate with bright excitonic PL emissions with high quantum yield and longer carrier lifetime that are conducive for high performance optoelectronic applications.^47–50^

Herein, through a systematic study of 13 pairs of *n* = 1 Pb and Sn iodide DJ perovskites sharing the same organic diammonium spacer cations, we examine why same-spacer Pb and Sn analogues can show markedly different emission line shapes and how this divergence can be converted into a design rule. To enable this comparison, we first synthesize and characterize four new DJ Sn perovskite crystal structures: (1,4CDMA)­SnI_4_, (1,3CDMA)­SnI_4_, (HDA)­SnI_4_, and (DGA)­SnI_4_, where 1,4CDMA = 1,4-cyclohexanedimethanammonium, 1,3CDMA = 1,3-cyclohexanedimethanammonium, HDA = hexyldiammonium, and DGA = dimethylbiguanide. Correlative analysis of the structure–property trends in this paired library of 26 total structures shows that favorable PL properties (narrow and symmetric PL peaks) in Sn DJ perovskites are associated with spacer cation environments that generate crystallographically equivalent perovskite pockets and suppress metal-site off-centering. We employ first-principles calculations and physics-constrained symbolic regression to identify an electron localization function (ELF) anisotropy descriptor that connects this structural motif to the directional stereochemical expression of the Sn^2+^ lone pair. Representative configuration-coordinate diagram calculations indicate that stronger lone-pair anisotropy is accompanied by larger excited-state reorganization. Following such design rules, a symmetric spacer cation, 2,3,5,6-tetrafluoro-1,4-phenylenedimethanammonium (4F-PDMA), is readily identified from reported Pb DJ perovskites to enable the synthesis and characterization of (4F-PDMA)­SnI_4_, which exhibits the expected structural parameters and favorable sharp and symmetric PL emission.

## Results and Discussion

### Divergent Photoluminescence in DJ Sn/Pb Perovskite Pairs

Because relatively few Sn DJ perovskites have been reported, most with poor photophysical properties, efficient strategies need to be developed to discover new phases with improved optoelectronic behaviors and sufficient air stability. We surveyed the ∼45 reported DJ lead iodide perovskite structures and identified four different (LA)­PbI_4_ phases that exhibit sharp PL and good optoelectronic properties, but their Sn DJ perovskite counterparts have not been reported. These four candidates are based on the following diammonium cations: dimethylbiguanide (DGA),^32^ 1,3-cyclohexanedimethylammonium (1,3CDMA),^51^ 1,4-cyclohexanedimethylammonium (1,4CDMA),^52^ and hexyldiammonium (HDA).^53^ By targeting the analogous Sn phases using typical aqueous hydroiodic acid solution growth,^11,29,38^ crystals of four new Sn DJ perovskites were synthesized and their crystal structures were determined using single-crystal X-ray diffraction, as shown in Figure 1a–d and Table S1 (see experimental details in Materials and Methods in the Supporting Information). We note that both (1,3CDMA)­SnI_4_ and (DGA)­SnI_4_ appear to be metastable and will transition to alternative black phases within an hour after removal from growth solutions.

**Figure 1:**
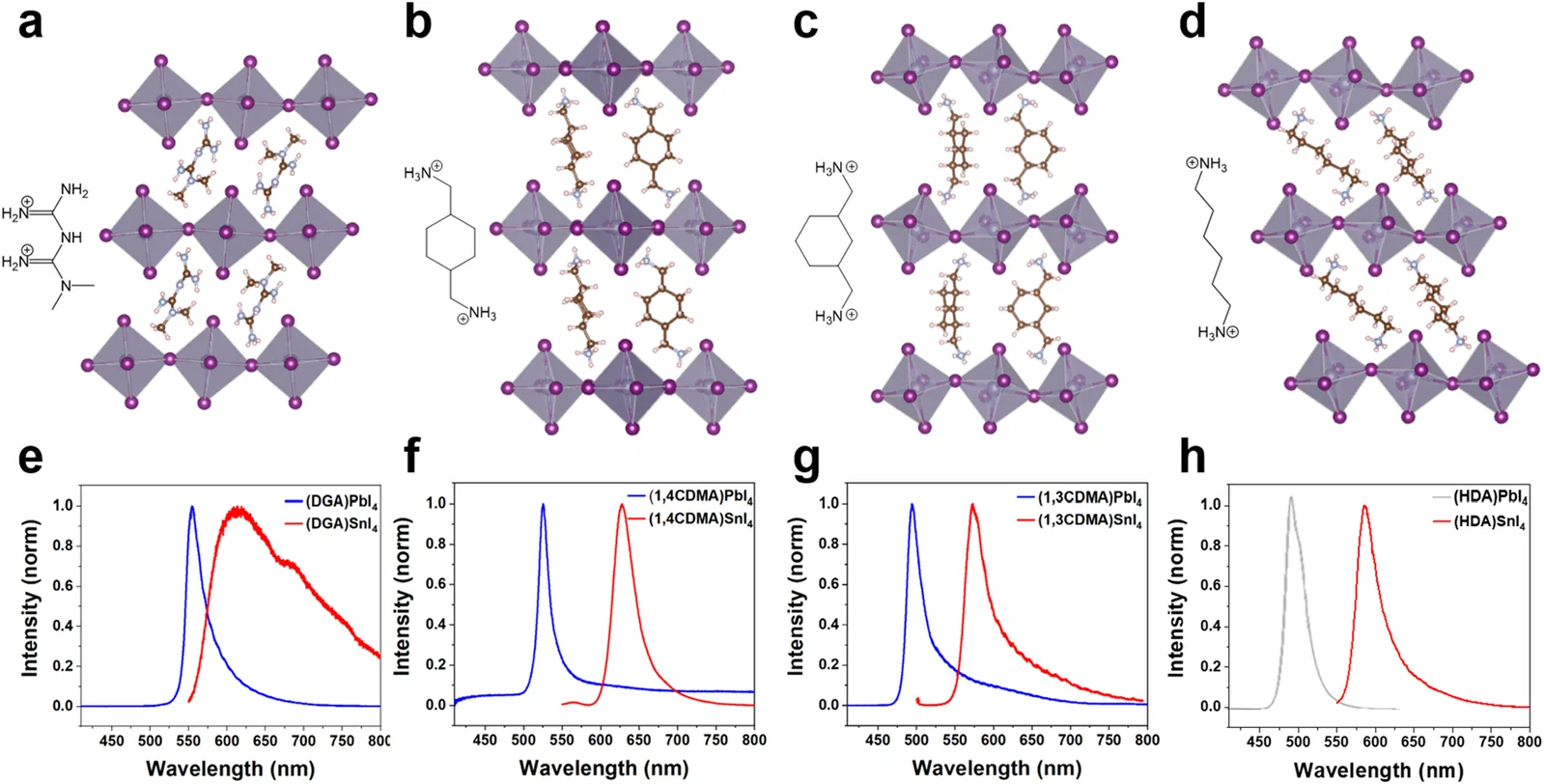
Crystal structures of new tin iodide DJ perovskites: (a) (DGA)­SnI_4_, (b) (1,4CDMA)­SnI_4_, (c) (1,3CDMA)­SnI_4_, and (d) (HDA)­SnI_4_, with the corresponding PL spectra (e–h, red curves) in comparison to the PL spectra of the corresponding Pb DJ perovskites (blue curves). The PL spectrum of (HDA)­PbI_4_ is reproduced from ref. 35

The PL spectra obtained for the exfoliated single-crystal flakes of each Sn DJ perovskite (Figure 1e–h, red curves) are compared with the PL spectra of the corresponding Pb phases (which were separately synthesized and characterized independently of previous reports, except for the PL spectrum of (HDA)­PbI_4_). Surprisingly, (DGA)­SnI_4_ exhibits a very broad and asymmetric PL emission, in a stark contrast to the sharp PL from (DGA)­PbI_4_ (Figure 1e),^32^ which was the reason the DGA cation was initially selected for exploration. In contrast, the PL characteristics of the new (1,3CDMA)­SnI_4_ and (1,4CDMA)­SnI_4_ are generally more similar to those of their corresponding Pb phases (Figure 1f,g): both Pb and Sn DJ perovskites exhibit relatively narrow peaks but the Sn perovskites show slightly broader peaks at red-shifted wavelengths. The PL line shape for (HDA)­SnI_4_ is also fairly narrow and symmetrical, quite similar to the PL spectrum of (HDA)­PbI_4_ (Figure 1h). Despite the previously reported synthesis,^53^ we found it difficult to reproducibly synthesize (HDA)­PbI_4_ against a more commonly formed competing non-2D phase, therefore the PL spectrum of (HDA)­PbI_4_ is reproduced from the literature.^36^ This divergence of PL property comparison between the Pb/Sn DJ perovskite pairs, especially the contrast between narrowband emitting (DGA)­PbI_4_ and broadband emitting (DGA)­SnI_4_, shows that sharp PL emission of Pb DJ perovskite alone is not sufficient to predict comparable narrow and symmetric PL emission in the analogous Sn DJ perovskite. This motivates a systematic structural comparison across Pb/Sn DJ analogue pairs.

### Structural Descriptors of 2D DJ Perovskite Frameworks

To investigate why the narrow and symmetric PL in DJ lead perovskites (suggestive of their excellent optoelectronic properties) do not always translate into similarly desirable PL characteristics in DJ tin perovskites based on the same spacer cations, we systematically compared the structural parameters and optical properties of a library of *n* = 1 DJ Pb and Sn iodide perovskites to parse out trends leading to different PL behaviors. At the time of search, there were less than 15 reported crystal structures of Sn iodide DJ perovskite deposited in the CCDC database and approximately 45 reported Pb iodide DJ perovskites. Of these reported structures, we only found 9 pairs of Pb/Sn DJ perovskite structures based on the same spacer cations that could be used for these systematic comparisons. Such pairings were limited due to a few factors. First, some Sn 2D DJ perovskites do not have analogous Pb DJ counterparts, but rather corrugated, non-2D lead iodide “perovskite-like” structures, such as for the pentanediammonium spacer cation.^54,55^ Second, only iodide-based structures were considered, so Pb/Sn bromide structures were not used for pairings. Finally, as a practical consideration, we chose DJ perovskites that are based on commercially available spacer cations so that we could easily synthesize both (LA)­BI_4_ phases and compare their PL properties under the same conditions. In addition to reported structures, the new Sn DJ perovskites we introduce in Figure 1 provide an additional four Pb/Sn pairs since the analogous Pb DJ perovskites were already reported. In total, 13 pairs of Pb/Sn DJ perovskites (26 crystal structures) were studied. These spacer cations are shown in Figure 2a together with their full chemical names and acronyms.

**Figure 2:**
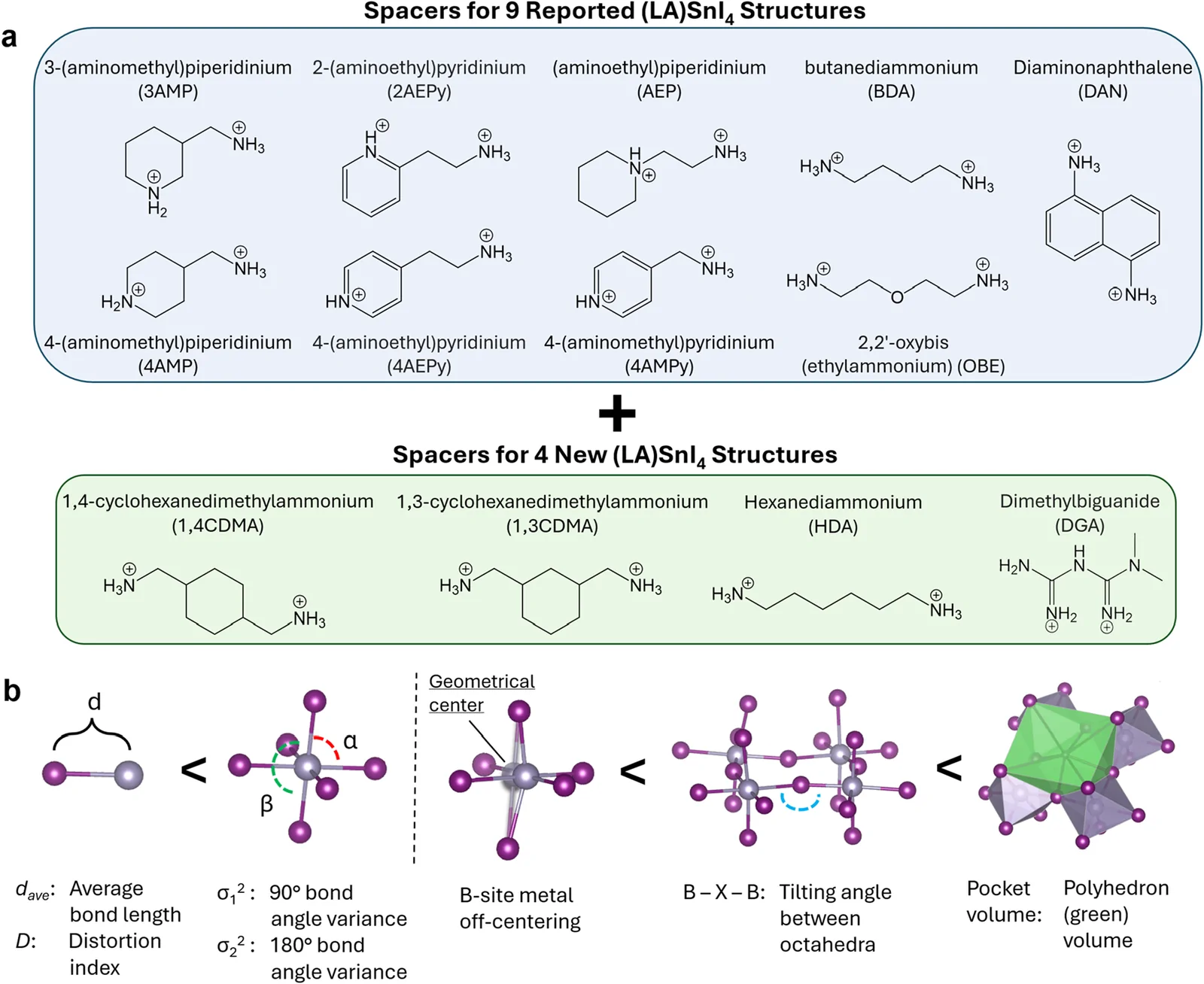
(a) Assembled library of reported and new spacer cations used to form pairs of *n* = 1 DJ tin and lead iodide perovskites studied herein. (b) Schematic illustrations of various structural parameters in *n* = 1 perovskite crystal structure with increasing levels of complexity, from B–I bond distortion < octahedral distortion and metal off-centering < octahedral tilting < pocket volume induced by spacer cation-lattice interactions.

We first assess the various structural parameters of each DJ perovskite. Structural distortion of the inorganic perovskite lattices from the ideal cubic geometrical structure can be described through a series of calculated structural parameters (Figure 2b). The average B–I bond length (*d*_ave_) is calculated along with the bond length distortion index (*D*), which is defined by $D=\frac{1}{6}\sum_{i=1}^{6}\left|\frac{d_{i}-d_{ave}}{d_{ave}}\right|$ . In an ideal perovskite structure with perfect [BI_6_] octahedra, *D* = 0 as all B–I bonds are the same length. Next, two angle distortion parameters of the [BI_6_] octahedra can be defined as the bond angle variance from the ideal 90 and 180° angles, where $\sigma_{1}^{2}=\frac{1}{11}\sum_{i=1}^{12}{\left(\alpha_{i}-90\right)}^{2}$ and $\sigma_{2}^{2}=\frac{1}{2}\sum_{i=1}^{3}{\left(\beta_{i}-180\right)}^{2}$ .^56^ For an ideal octahedron, σ_1_^2^ and σ_2_^2^ are both 0. Separately, we also calculated B-site metal off-centering displacement, which is defined as the distance between the metal center and the geometrical center of the six iodine atoms.^57^ The tilting between octahedra that form a 2D perovskite layer can be described by the average B–I–B angle (which deviates from 180° in a nonideal structure). Finally, 2D perovskite structures exhibit a distinct pocket volume to accommodate the ammonium heads of the spacer cations, which is defined as the polyhedral volume formed between four bridging and four terminal iodides (Figure 2b, green polyhedron).^47,49^ These structural distortion parameters and the pocket volume are influenced by the interaction of the spacer cations and the inorganic lattice through ammonium headgroup penetration and sterics.^20,48,49,58^ We note that some structures exhibit more than one unique BI_6_ octahedra, tilting angle, etc., (most often two, but sometimes four, symmetrically related). In these cases, averages of the above parameters are calculated. These structural parameters, as well as the interlayer spacing, were determined for the 26 DJ Pb and Sn structures and summarized in Table 1 (the CCDC codes for the structures analyzed can be found in Table S2). Notably, Sn DJ perovskites exhibit a wider range of structural distortion than Pb DJ perovskites (Figure S1). This is consistent with the previous observation that Sn RP perovskites exhibit a wider variation in bond lengths than their Pb analogues.^59,60^ These observations suggest that Sn-based DJ lattices can accommodate more severe local structural distortions than Pb analogues, which may help explain both the structural diversity and the stronger sensitivity of photophysics to local distortion environments in Sn perovskites.

### Correlating Structural and Emission Trends across Library of Sn/Pb DJ Perovskite Pairs

To better understand the structure-PL correlations in DJ perovskites, we generated 2D correlation plots using various combinations of structural parameters from Table 1, with the Pb and Sn structures plotted together and color coded by spacer cation. The *D* vs σ_1_^2^ plot (Figure 3a) provides the most intuitive representation of the unique behaviors of different phases. Arrows are used to highlight the changes in structural parameters from the Sn (square) to Pb (triangle) DJ perovskite structures based on the same spacer cations. Five spacer pairs, (BDA)­BI_4_, (HDA)­BI_4_, (DAN)­BI_4_, (1,3CDMA)­BI_4_, and (1,4CDMA)­BI_4_ form a compact cluster (highlighted by a red circle) in which the Pb and Sn analogues exhibit similarly low structural distortions. This clustering of structures is also apparent in several other descriptor combinations, including *D* vs σ_2_^2^, *D* vs B–I–B angle, *D* vs pocket volume, σ_1_^2^ vs σ_2_^2^, and σ_1_^2^ vs B–I–B angle (Figure S2). In contrast, Pb/Sn pairs based on the other spacer cations not only exhibited greater structural distortions, but also more significant differences between the Pb versus Sn structures across the parameter ranges.

To understand if the observed structural correlations of Pb/Sn pairs also correlate with their PL characteristics, we tried to synthesize Pb and Sn iodide perovskites based on all 13 spacer cations through the conventional slow cooling growth in hydroiodic acid (Figure S3). Some difficulty was encountered in obtaining some of the reported phases. (BDA)­PbI_4_ has a competing, corrugated perovskite crystal phase^69^ that was repeatedly obtained as opposed to the 2D DJ structure. (4AMPy)­PbI_4_ is known to be difficult to synthesize^38^ and could not be obtained for this comparison, just as for the (HDA)­PbI_4_ mentioned previously. However, the majority of the perovskite crystals were obtained for structure–property comparisons. Since the potential impurity phases do not have 2D perovskite structures and exhibit emission or absorption with much shorter wavelength, the successful synthesis of these DJ perovskites can be readily verified by the expected dark red-to-black crystal color (Figure S3) or expected PL emission wavelength. Select powder X-ray diffraction (PXRD) patterns of synthesized crystals are shown in Figure S4, confirming the synthesized crystals matched the analyzed crystal structures.

PL spectra were measured on multiple exfoliated flakes for each phase so that average PL properties could be determined (see Table S3 for the numbers of objects measured). A representative PL trace for each perovskite can be seen in Figures S5 (Sn) and S6 (Pb). Many Pb-based DJ perovskites consistently show a lower energy peak (4AMP, 2AEPy, 4AEPy, AEP), although the excitonic emission peak remains quite narrow.^70^ The nature of this low energy peak is highly debated in perovskite literature and has been frequently seen in DJ perovskites, although commonly attributed to defects,^71,72^ it could also be caused by self-trapped excitons and edge effects.^73,74^ In contrast, Sn DJ perovskites rarely exhibit this second, low energy emission, but often exhibit overall broadband emission (DGA, AEP, OBE). Interestingly, the center PL emission wavelengths for these *n* = 1 (LA)­SnI_4_ DJ structures span approximately 100 nm (0.3 eV), a much larger variation than that observed in most RP perovskites.^75^ We calculate two parameters from each PL spectrum: Full Width at Half Maximum (FWHM) and Asymmetric Factor (AF), as illustrated in Figure 3b.^47^ FWHM has been a common descriptor for bright emission and higher PL quantum yield (PLQY).^48,59^ AF highlights the elongated red tail of emission associated with electron–phonon coupling in perovskites.^47,59,76,77^ The averages and standard deviations of FWHM and AF across multiple PL spectra for each phase are summarized in Figure S7. We observe that the FWHMs for Pb DJ perovskites are always narrower than their corresponding Sn phase. Meanwhile, there is no discernible trend between the AF of the Pb and Sn DJ perovskite pairs, although the strong second emission peaks of the Pb DJ perovskites cause high standard deviations of the AF, which could obscure the true AF. We note that these analyses are performed in energy scale as the conversion between wavelength and energy scale is nonlinear so broader peaks may be overexaggerated.^78^ Performing the same spectral analyses in wavelength scale shows very close agreement in the FWHM trend across most phases, but some discrepancy is observed in the AF factor for broad emitting phases ((DGA)­SnI_4_, (2AEPy)­SnI_4_, (AEP)­SnI_4_, and (OBE)­SnI_4_): the AF analyzed in the energy scale appears more symmetrical (Figure S8). Henceforth, we qualitatively describe PL with smaller FWHM and AF close to 1 as having favorable line width and line shape characteristics, and broader and/or less symmetric emission as less favorable.

Presenting Figure 3a in 3D plots using these PL metrics (FWHM and AF) as the column height (Figure 3c,d) shows that the clustered region of the plots contains the Sn DJ perovskite phases with the most favorable line width and line shape metrics. The shared FWHM and AF height scales between Pb and Sn phases emphasizes that Sn DJ perovskites generally exhibit broader and/or less symmetric emissions than their Pb analogues, similar to what was shown in Figure S7. We note that in the clustered region, due to the close proximity, the perovskite phases with the narrowest FWHM and most symmetrical AF are obscured in these 3D plots due to being the shortest columns. These correlation plots reveal an empirical window of structural parameters associated with narrower, more symmetric PL emission for DJ Sn iodide perovskites: *D* = 0–0.1, σ_1_^2^ = 3–6, σ_2_^2^ = 0, metal off-centering = 0, and B–I–B = 146–158°, and relatively small pocket volumes (Figure S2 and Table 1). These five pairs of DJ perovskites in the clustered regions, (BDA)­BI_4_, (HDA)­BI_4_, (DAN)­BI_4_, (1,3CDMA)­BI_4_, and (1,4CDMA)­BI_4_, consistently exhibit low octahedral distortion with low σ_1_^2^ and zero B-site off-centering for all five structures. The representative narrow and symmetric PL spectra from these five Sn DJ iodide perovskites are shown in Figure 3e.

Interestingly, these clustered DJ perovskite phases with narrow and symmetric PL character should not be interpreted as having globally undistorted inorganic lattices. While they exhibit low intraoctahedral distortions, they also exhibit substantial interoctahedral tilting, as reflected in their B–I–B angles (146–158°). These B–I–B angles tend to be more tilted (*i*.*e*. further from 180°) than the other Sn DJ structures that exhibit greater intraoctahedral distortions, B-site off-centering, and poorer PL qualities (Figure S2b). This distinction is important because octahedral tilting and metal off-centering can act as separable distortion channels in halide perovskites.^79,80^ The narrowband emitting Sn DJ perovskites therefore appear to tolerate octahedral tilting of the perovskite framework while minimizing the local metal off-centering most closely associated with Sn lone-pair anisotropy. Unlike previous reports on RP perovskites,^47,59^ we do not find a strong correlation between the average bond length (*d*_ave_) and the PL quality of DJ perovskites.

Since spacer cation is the only organic moiety that could be varied in *n* = 1 DJ perovskites, the spacer cation molecular structures therefore must control the crystallographic features observed above. For many spacer cations with a ring-and-chain motif, such as AMP and AEPy (see Figure 2a), there are at least two crystallographically distinct perovskite pocket volumes to accommodate the differently sized ammonium head groups, one for the ring end and one for the chain end (Figure 3f left side and Table S4). DJ perovskites using these asymmetric spacers tend to exhibit broad PL emission (Figure S5). In contrast, each of the five DJ perovskites with narrow and symmetric PL character in the clustered set utilize spacer cations with high molecular symmetry (BDA, HDA, DAN, 1,3CDMA, and 1,4CDMA), i.e., their two ammonium heads are symmetrical about mirror planes and/or inversion centers (see Figure 2a). This feature appears to frequently lead to DJ perovskite structures with crystallographically equivalent perovskite pockets with the same pocket volume (illustrated in Figure 3f right side), which are only observed for spacer cations in the clustered group. Interestingly, although OBE is also symmetrical, there are two different pocket volumes in both the Pb and Sn perovskite structures, indicating two different interactions between this spacer cation and the lattice at each end of the molecule (Table S4). This further highlights that the OBE spacer cation does not fall within the parameter set that correlates with narrow and symmetric PL despite its molecular symmetry and corroborates the poor PL emission displayed by (OBE)­SnI_4_ (Figure S5). Thus, perovskite pocket volume equivalence also emerges as a useful structural marker for predicting narrow and symmetric PL emissions in DJ perovskites. Additionally, we note that despite using averaged structural parameters to construct the correlation plots, structures that have multiple octahedra can still fall within the requirements of zero metal off-centering and equivalent pocket volumes (such as (BDA)­SnI_4_), strengthening this trend despite the limited phase space.

**Table 1:** Calculated Structural Parameters for the 13 Pairs of Sn (Top) and Pb (Bottom) DJ Iodide Perovskite Structures in This Study

	1,3CDMA^51^	1,4CDMA^52^	HDA^53^	DGA^32^	3AMP^14,30^	4AMP^30,61^	BDA^62,63^	2AEPy^60,64^	4AEPy^60,64^	4AMPy^38,65^	AEP^54,66^	OBE^67,68^	DAN^29,62^
space group	*Pnma*	*P*2_1_/*c*	*P*2_1_/*c*	*P*2_1_/*c*	*P*4	*Pca*2_1_	*P*-1	*P*2_1_/*c*	*P*2_1_/*c*	*Pmmn*	*P*2_1_/*c*	*P*2_1_/*c*	*P*2_1_/*c*
d_ave_ (Å)	3.162	3.152	3.157	3.205	3.129	3.169	3.156	3.170	3.150	3.158	3.205	3.166	3.169
*D*	0.00509	0.00421	0.00550	0.0493	0.00362	0.0215	0.00206	0.0443	0.0325	0.00192	0.0478	0.0399	0.00989
σ_1_^2^	3.431	5.130	3.921	27.284	8.158	15.144	3.225	37.510	17.980	0.00430	26.298	14.725	5.372
σ_2_^2^	0	0	0	136.8	22.4	26.0	0	114.2	29.0	0	60.1	47.2	0
off centering (Å)	0	0	0	0.2540	0.0613	0.1002	0	0.1955	0.0612	0	0.1113	0.1300	0
B–X–B (°)	148.6	157.8	150.5	169.1	169.5	157.4	150.4	163.5	167.9	151.8	171.7	160.4	152.2
pocket volume (Å^3^)	92.53	99.39	93.38	108.95	107.36	103.85	94.04	101.96	101.38	97.87	107.48	104.63	98.26
interlayer spacing (Å)^a^	6.365	5.867	5.049	3.763	4.067	4.132	4.024	3.661	4.040	4.130	4.201	3.682	4.921
													
space group	*Pna*2_1_	*P*2_1_/*c*	*P*2_1_/*c*	*P*2_1_/*c*	*P*2_1_/c	*Pc*	*C*2/*c*	*P*2_1_/*c*	*P*2_1_/*c*	*Pc*	*P*2_1_/*c*	*P*2_1_/*c*	*P*2_1_/*c*
d_ave_ (Å)	3.208	3.186	3.200	3.196	3.182	3.202	3.201	3.196	3.180	3.197	3.208	3.196	3.212
*D*	0.00485	0.00473	0.00530	0.00713	0.00455	0.0191	0.00198	0.0133	0.0129	0.00593	0.0141	0.0141	0.00887
σ_1_^2^	4.415	6.771	3.301	20.710	17.031	9.480	3.825	26.181	16.726	0.692	37.639	10.571	7.172
σ_2_^2^	0.0216	0	0	111.1	14.5	50.6	0	70.8	24.0	3.54	127.2	23.9	0
off centering (Å)	0	0	0	0.0687	0.0449	0.0740	0	0.1045	0.0443	0.0376	0.1028	0.0677	0
B–X–B (°)	146.1	153.5	148.3	173.6	165.1	154.4	147.1	164.5	168.1	149.1	169.9	158.6	150.6
pocket volume (Å^3^)	94.94	100.73	96.06	107.62	104.20	102.74	96.06	104.74	103.93	99.73	112.4	105.8	101.39
interlayer spacing (Å)^a^	5.720	5.720	5.103	3.730	3.816	4.175	4.018	3.709	4.175	4.161	3.908	3.700	4.856

a Interlayer spacing is defined as the distance between the planes formed by terminal iodides.

### Electronic Structure Origins of Photoluminescence Line shape Broadening

The structural analysis identifies low metal off-centering and pocket equivalence as key empirical markers, but it does not by itself reveal how these motifs reshape the electronic structures and, in turn, the emission properties of DJ perovskites. We therefore performed density functional theory (DFT) calculations on three representative DJ perovskites: narrowband emitting (1,4CDMA)­SnI_4_ as a member of the clustered set of DJ Sn perovskites, broadband emitting (DGA)­SnI_4_ as a highly distorted Sn outlier, and narrowband emitting (DGA)­PbI_4_ as a reference Pb DJ perovskite based on the same spacer cation. Band structure analyses and partial charge-density visualizations of the valence band edge show that (1,4CDMA)­SnI_4_ has a nearly isotropic Sn-centered charge distribution (Figure 4a) and relatively dispersive band-edge states (Figure S9a). In contrast, (DGA)­SnI_4_ shows anisotropic charge distribution indicative of its directional Sn 5s^2^ lone-pair expression (Figure 4b) and flatter band-edge dispersion (Figure S9b). With the same DGA spacer cation, (DGA)­PbI_4_ shows weaker Pb 6s^2^ lone-pair anisotropy (Figure 4c). This Pb/Sn contrast is chemically reasonable because the Sn 5s^2^ lone pair is less relativistically stabilized than the Pb 6s^2^ lone pair, making the Sn-centered electronic structure more sensitive to local symmetry breaking.^56,59^

To better understand how local structure influences electronic properties, we analyzed one-dimensional electron localization function (ELF) profiles along the six Sn–I coordination directions of each SnI_6_ octahedron (Figure S10). For each direction i, we extracted the position *r*_*i*_ and height *h*_*i*_ of the first Sn-centered valence-shell maximum and combined these quantities with the Sn–I distance *d*_*i*_ as the primary inputs to a physics-constrained SISSO++ symbolic-regression analysis (see Supporting Information and Table S5 for details).^81,82^ The search identified a compact, physically interpretable descriptor of directional ELF anisotropy. For direction i at inequivalent Sn site j, *M*_*ij*_ = *r*_*ij*_ *h*_*ij*_; the site-level anisotropy is *A*_*j*_ = [max_*i*_(*M*_*ij*_) – min_*i*_(*M*_*ij*_)]/mean_*i*_(*M*_*ij*_), and the material-level amplitude is *A*_ELF_ = max_*j*_(*A*_*j*_). Lower *A*_ELF_ indicates a more isotropic electron-localization environment, whereas higher *A*_ELF_ reflects more directional lone-pair anisotropy. The final identified descriptor, *A*_ELF_^2^, correlates closely with the measured PL FWHM across the 13 Sn DJ perovskites (*R* ^2^ = 0.907; Figure 4d), capturing the overall progression from low ELF anisotropy and narrow PL to higher ELF anisotropy and broader PL. The strength of this relationship indicates that the static lone-pair anisotropy already captures the dominant cross-material variation in PL line width, despite the fact that finite-temperature lone-pair dynamics are not explicitly included.^83,84^ Therefore, the ELF anisotropy descriptor provides an electronic descriptor of Sn-centered lone-pair directionality that is consistent with the structural descriptors of metal off-centering/pocket equivalence and more directly connected to PL line shape broadening.

**Figure 3:**
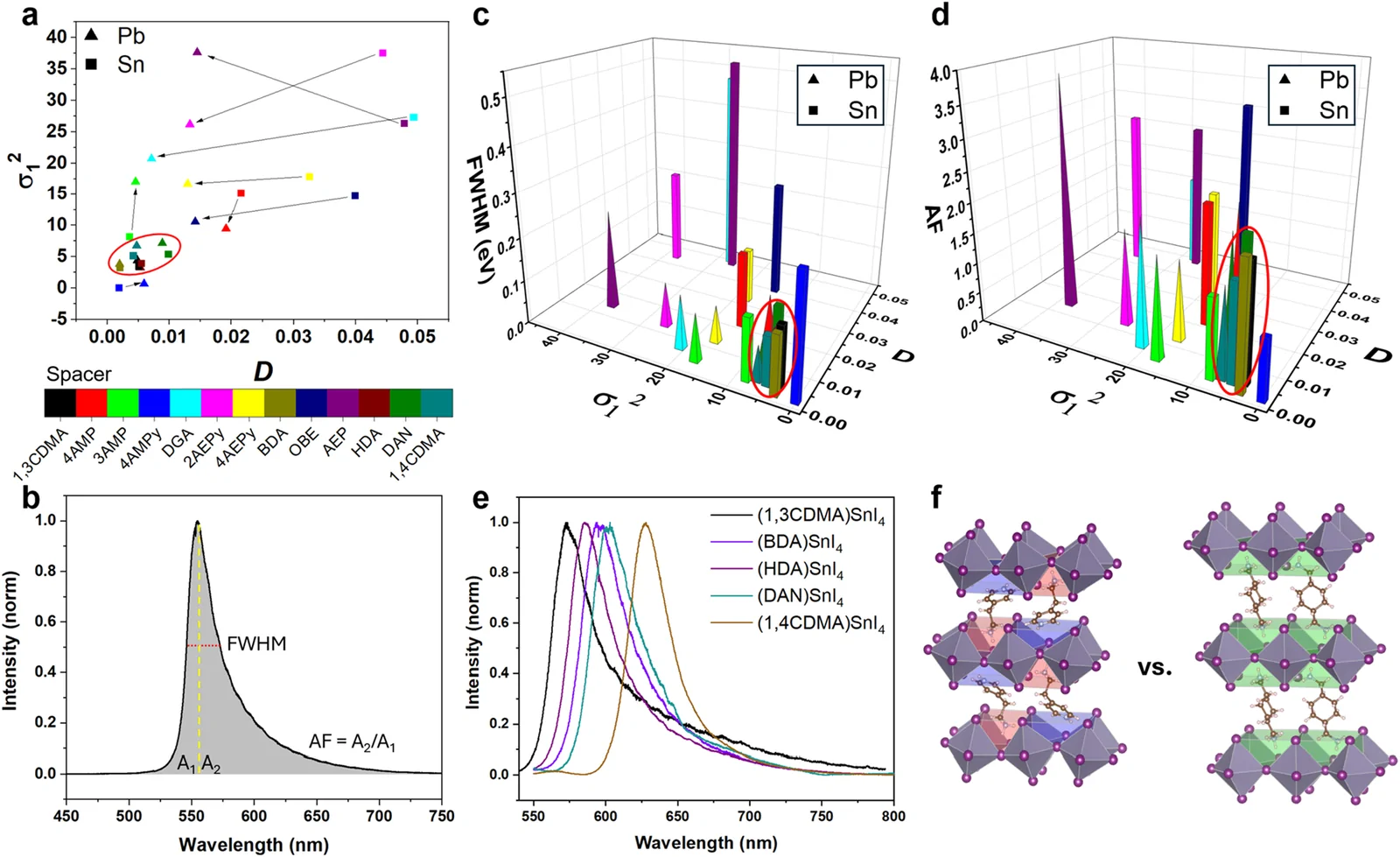
Clustering of structural parameters and PL characteristics in the library of Sn/Pb DJ perovskite pairs. (a) Structural correlation plot of *D* vs σ_1_^2^ of the DJ Pb (triangles) or Sn (square) iodide perovskites with color mapping by spacer cations. Red circle highlights the set of clustered Pb/Sn perovskites. (b) Illustration showing how FWHM and AF are calculated from a measured PL spectrum. Note that the reported FWHM and AF values are calculated in the energy scale (eV). (c, d) Three-dimensional (3D) correlation plots of *D* vs σ_1_^2^ with column height representing the (c) FWHM (eV) and (d) AF parameters of the PL emission. Color mapping by spacer cation same as plot (a). Clustered Pb/Sn DJ perovskites highlighted by red ellipsoid (small columns within the cluster are obscured). These plots show that the clustered Pb/Sn DJ perovskites also exhibit relatively narrow and symmetric PL emission. (e) PL spectra of the five clustered Sn DJ iodide phases that show relatively narrow and symmetric PL emission. (f) Visualization of the different symmetries of pocket volume (blue/pink and green polyhedra) for either end of the diammonium spacer cations: alternating pocket volumes (using the example of (4AEPy)­SnI_4_) versus identical pocket volumes (using the example of (1,3CDMA)­SnI_4_).

These calculations additionally clarify the distinction between two structural descriptors which are often conflated in the halide perovskite literature. The bond-angle variance σ_2_^2^ describes angular distortion along the 180° angle of the BI_6_ octahedron, whereas metal off-centering measures displacement of the B-site metal cation from the geometrical center of the surrounding iodides. These two descriptors often correlate because angular distortion and B-site displacement typically occur together in distorted perovskite lattices, as shown for the 26 Pb/Sn DJ perovskites herein in Figure S11. However, this correlation does not always hold true.^47^ For example, (DGA)­PbI_4_ exhibits a large σ_2_^2^ value of 111.1 but low metal off-centering, and subsequently low ELF anisotropy (Figure S12). This exception shows that a highly distorted octahedral angle environment does not necessarily imply strong B-site lone-pair anisotropy. Instead, the clearer correlation between metal off-centering and ELF anisotropy indicates that metal off-centering is the more direct structural proxy for stereochemically active lone-pair directionality. This distinction is important for comparing Pb and Sn DJ perovskites, because Sn-centered electronic structure is more sensitive to local symmetry breaking and therefore requires a more precise descriptor tied directly to metal-site displacement rather than to octahedral angular distortion alone.

**Figure 4:**
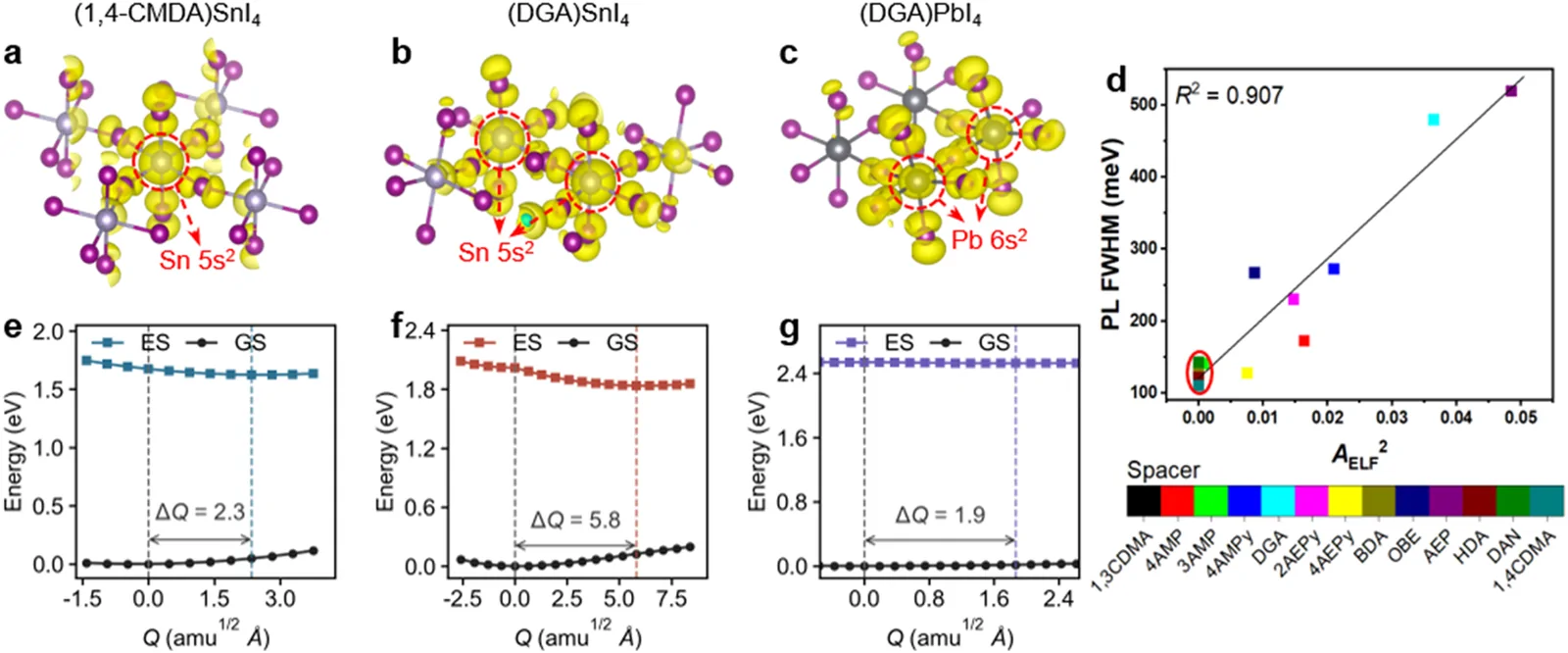
Electronic structure origins of PL line shape broadening in DJ perovskites. (a–c) Representative lone-pair charge-density visualizations for the (a) narrowband emitting (1,4CDMA)­SnI_4_, (b) broadband emitting (DGA)­SnI_4_, and (c) the corresponding Pb analogue (DGA)­PbI_4_ with sharp PL emission. These comparisons show weak Sn-centered 5s^2^ lone-pair anisotropy in the (1,4CDMA)­SnI_4_, strong Sn lone-pair anisotropy in (DGA)­SnI_4_, and weaker Pb 6s^2^ lone-pair anisotropy under the same spacer environment. (d) Physically interpretable ELF-anisotropy descriptor (*A*_ELF_^2^), identified by SISSO++ symbolic regression, plotted against the measured PL FWHM for the Sn DJ perovskites, which are colored corresponding to spacer cations. The red ellipse highlights the five clustered Sn DJ perovskites with low *A*_ELF_^2^ and narrow PL line widths. (e–g) Configuration-coordinate diagrams for parameters for (e) (1,4CDMA)­SnI_4_, (f) (DGA)­SnI_4_, (g) (DGA)­PbI_4_. Vertical dashed lines mark the ground-state and excited state minima, and arrows indicate the generalized displacement Δ*Q*.

Furthermore, we examined how this ground-state lone-pair anisotropy is transmitted to excited-state photophysics. In a pseudo-Jahn–Teller framework, stronger stereochemical activity of the Sn^2+^ 5s^2^ lone pair is associated with a softer local potential along an off-centering coordinate, which can facilitate structural relaxation after excitation.^56,59,85^ We therefore performed configuration-coordinate diagram (CC diagram) calculations of the emission process for the representative phases (Figure 4e–g). From narrowband emitting (1,4CDMA)­SnI_4_ to broadband emitting (DGA)­SnI_4_, the fitted Huang–Rhys factor increases from 5.85 to 17.1, indicating stronger electron–phonon coupling and greater excited-state reorganization in the Sn DJ perovskites with stronger ELF anisotropy. In contrast, the control Pb DJ perovskite with the same spacer cation, (DGA)­PbI_4_, shows a much smaller Huang–Rhys factor of 2.48 and a smaller excited-state displacement, consistent with weaker Pb-centered ELF anisotropy. These trends indicate that stronger ground-state metal lone-pair anisotropy is accompanied by greater excited-state structural reorganization and electron–phonon coupling, providing a plausible route to increase vibronic broadening of PL emission.

This explanation is consistent with the experimental photophysics interpretation. Although self-trapped excitons (STE) are often invoked to explain low-energy broadband emission in distorted lead bromide and germanium perovskites,^49,86–88^ all DJ tin iodide perovskites studied here emit in the same general spectral region and show relatively small Stokes shifts based on their absorption spectra (Figure S13 and Table S6). This suggests that distortions of DJ tin iodide perovskites does not result in STE, but instead broaden the free excitonic (FE) emission due to metal off-centering.^50^ Low-temperature PL of (DGA)­SnI_4_, (OBE)­SnI_4_, and (AEP)­SnI_4_ further show a narrowing of the excitonic emission peak (Figure S14), similar to the behaviors reported for other DJ Sn perovskites and supporting a substantial electron–phonon contribution to the PL line width at room temperature.^32,89–93^ Occasionally, we observe a broad emission emerge at low temperatures for (AEP)­SnI_4_, which might be attributed to STE.^92^ Therefore, all of these theoretical and experimental results suggest that the broad PL emissions of these Sn DJ perovskites are most consistently described as vibronically broadened excitonic emission associated with lone-pair-driven excited-state relaxation, rather than as a distinct STE band.

### Designing a New 2D DJ Tin Iodide Perovskite with Narrow PL Emission

All of our results support a mechanistic design rule underlying the structural screening criteria: organic spacer cation environments that preserve equivalent inorganic perovskite pockets suppress Sn-centered lone-pair anisotropy, thus limiting excited-state reorganization and electron–phonon broadening and improving the PL characteristics of DJ perovskites. We will now demonstrate the use of these structural screening criteria and design rules to quickly identify a new spacer cation candidate for DJ tin perovskites. Because narrow and symmetric PL emission from Pb DJ perovskite does not guarantee favorable PL emission for analogous Sn DJ perovskite, reported Pb DJ structures are not used as direct proxies for PL properties, but as a spacer-geometry library for candidate Sn analogues. We applied the structural criteria (i.e., the desired window of structural parameters) above to prospectively screen the more than 30 reported Pb DJ perovskites that lack Sn counterparts, prioritizing symmetrical spacer cations expected to minimize metal off-centering and generate equivalent inorganic pocket environments, while limiting to commercially available reagents. From these 30 candidates, we quickly identified a multifluorinated aromatic spacer cation, 2,3,5,6-tetrafluoro-1,4-phenylenedimethanammonium (4F-PDMA), which has been utilized in Pb-based 2D/3D perovskite solar cell devices exhibiting high environmental stability and high resistance to ion diffusion.^35,94^ Structural analysis of the identified (4F-PDMA)­PbI_4_ shows that is displays zero metal off-centering and equivalent pocket volumes, making 4F-PDMA an ideal spacer cation candidate from which to synthesize the analogous Sn perovskite.

Using this identified 4F-PDMA spacer cation, we synthesized a new (4F-PDMA)­SnI_4_ DJ perovskite and determined its crystal structure (Figure 5a, crystallographic details in Table S1). Interestingly, the benzene rings of neighboring 4F-PDMA spacers are not π–π stacked, which was previously argued to not be conducive for the formation of stable DJ perovskites.^37^ Such packing motif of 4F-PDMA is likely caused by the interactions of multiple F atoms around the benzene ring. We note that the Pb analogue (4F-PDMA)­PbI_4_ exhibits the narrowest FWHM, the most symmetric emission, and the most blue-shifted PL emission (Figure 5b blue curve) among the measured Pb DJ perovskites in the library herein. As expected, the analogous (4F-PDMA)­SnI_4_ exhibits one of the narrowest and most symmetric PL emission profiles (Figure 5b red curve) among all Sn DJ perovskites studied herein. The PL emission wavelength of (4F-PDMA)­SnI_4_ aligns with the other Sn DJ perovskites (Figure S15), unlike the significantly blue-shifted PL observed for the Pb analogue. Both the (4F-PDMA)­PbI_4_ and (4F-PDMA)­SnI_4_ phases fall within the favorable clustered region previously identified (Figure 5c) and exhibit very similar structural parameters (low structural distortion and equivalent perovskite pockets) as well as small FWHM and low AF of PL emissions (Figure S16). This comparison, which was made possible after (4F-PDMA)­SnI_4_ was synthesized by following solely our proposed design criteria and then crystallographically characterized, reinforces that the design success arises from the spacer-controlled structural parameter window rather than a direct Pb-to-Sn analogy. DFT calculations further place (4F-PDMA)­SnI_4_ in the low ELF-anisotropy regime (*A*_ELF_^2^ = 6.15 × 10^–6^) characteristic of Sn DJ perovskites with narrow and symmetric PL character. Its CC diagram response shows a Huang–Rhys factor of 8.97 and generalized displacement of 2.90 amu^1/2^ Å (Figure S17), which falls between the narrowband emitting (1,4CDMA)­SnI_4_ and broadband emitting (DGA)­SnI_4_ reference phases (see Figure 4e,f).

**Figure 5:**
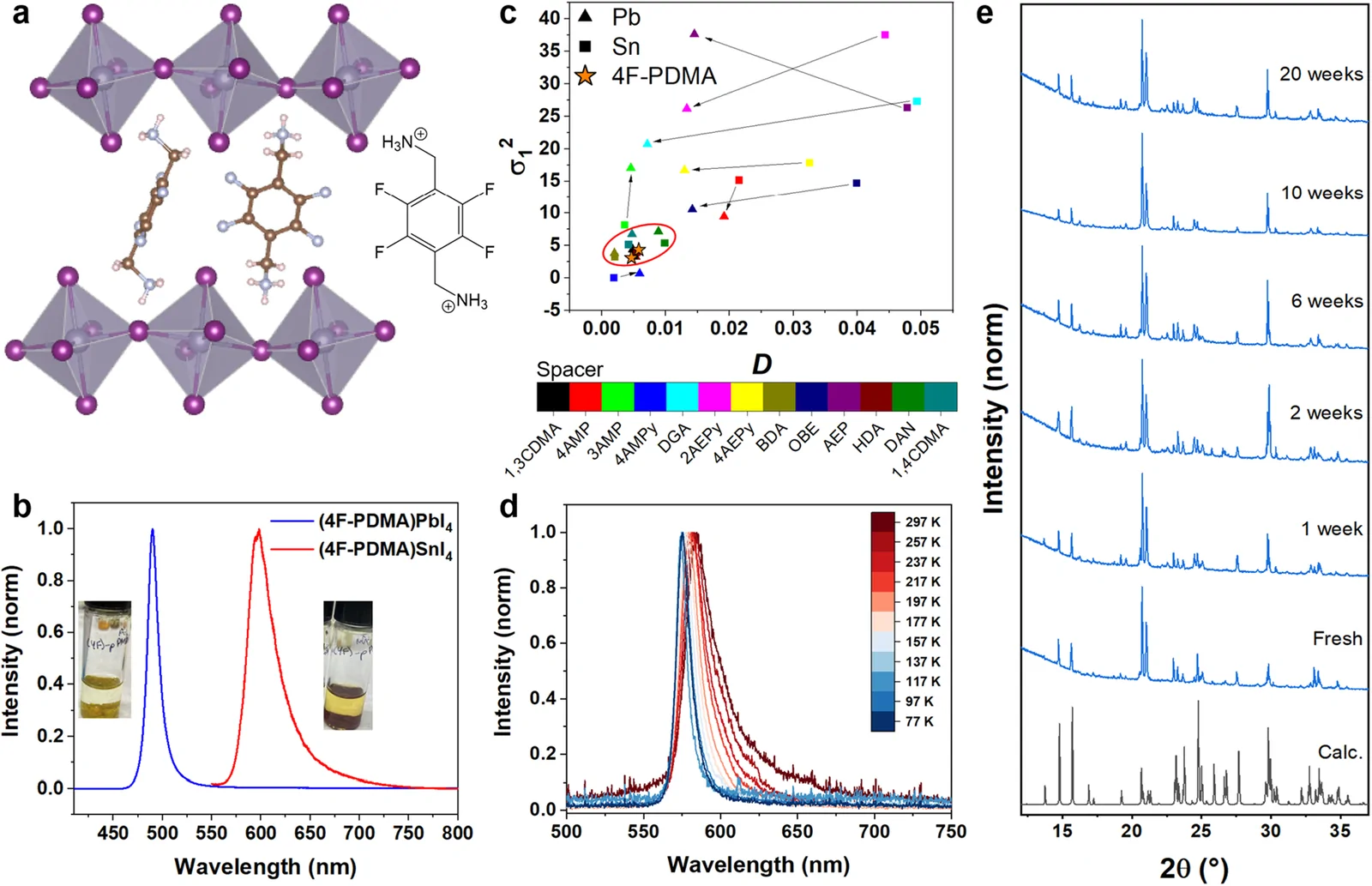
Synthesis and properties of new (4F-PDMA)­SnI_4_ perovskite. (a) Experimentally determined crystal structure of (4F-PDMA)­SnI_4_ and chemical structure of the 4F-PDMA spacer cation. (b) PL spectra of (4F-PDMA)­PbI_4_ (left) and (4F-PDMA)­SnI_4_ (right) with accompanying photographs of synthesized crystals in vials. (c) Structural correlation plot of *D* vs σ_1_^2^ of the DJ Pb (triangles) or Sn (square) iodide perovskites with color mapping by spacer cations following Figure 3a with the (4F-PDMA)­PbI_4_ and (4F-PDMA)­SnI_4_ added as orange stars. Red circle indicates the clustered region of DJ perovskites with favorable PL characteristics. (d) Temperature-dependent PL spectra of (4F-PDMA)­SnI_4_. (e) PXRD patterns of (4F-PDMA)­SnI_4_ calculated from crystal structure (black trace) and experimental patterns (blue traces) over the course of 20 weeks in ambient conditions (relative humidity = 25–50%), demonstrating excellent bulk stability.

Temperature dependent PL spectra of (4F-PDMA)­SnI_4_ also show a narrowing of the emission peak as temperature decreases (Figure 5d), although to a lesser degree than those broad emitting DJ Sn phases (Figure S14). The FWHM as a function of temperature was plotted for both (4F-PDMA)­SnI_4_ and (DGA)­SnI_4_, as examples of a narrowband and broadband emitter, respectively (Figure S18). These data were fit according to the equation Γ­(*T*) = Γ_0_ + Γ_LO_ = Γ_0_ + γ_LO_/(e^*E*_LO_/*k*_B_ *T*^–1), where Γ_0_ is the inhomogeneous broadening term, Γ_LO_ is the homogeneous broadening term due to Fröhlich longitudinal optical (LO) phonon scattering, γ_LO_ is the charge-carrier-phonon coupling strength, and *E*_LO_ is the phonon energy.^32,92,95,96^ The fitted parameters of electron–phonon coupling strength are summarized in Table S7 together with the values reported for other DJ Pb and Sn iodide perovskites. Importantly, among the tabulated Sn DJ iodide perovskites, the newly reported (4F-PDMA)­SnI_4_ exhibits the lowest value of γ_LO_ = 303.2 meV, consistent with its narrow PL emission. In contrast, (DGA)­SnI_4_ exhibits the largest γ_LO_ value of 1367 meV, consistent with its much broader emission. These results further qualitatively support the earlier interpretation of vibronically broadened excitonic emission associated with lone-pair-driven excited-state relaxation in these Sn DJ iodide perovskites.

Moreover, (4F-PDMA)­SnI_4_ exhibits excellent bulk stability in ambient air and does not show noticeable bulk degradation over 20 weeks based on periodic monitoring of PXRD patterns (Figure 5e). This is likely due to the DJ-type spacer cation that is halogenated, which are two spacer characteristics shown to increase stability in Sn perovskites.^29,97^ Taken together, the low structural distortions, low ELF anisotropy, intermediate CC diagram response, and narrow, relatively symmetric PL of (4F-PDMA)­SnI_4_ provides a verification of the structural and electronic screening criteria toward the design of high performance (and stable) Sn DJ perovskites. The agreement between these structural and optoelectronic parameters across several DJ perovskites supports perovskite pocket volume equivalence and suppressed Sn lone-pair anisotropy as practical criteria for identifying Sn DJ perovskites with narrow and symmetric PL emission.

## Conclusion

In summary, a systematic study of 13 Pb and Sn iodide DJ perovskite pairs that share the same spacer cations yields a design framework for *n* = 1 Sn DJ perovskites with favorable narrow and symmetric PL emissions. We report 5 new *n* = 1 Sn iodide DJ perovskite structures based on the spacer cations of 1,3CDMA, 1,4CDMA, HDA, DGA, and 4F-PDMA, greatly expanding the pool of reported Sn iodide DJ perovskites. The DJ perovskites with the most favorable PL characteristics share two structural features: negligible metal off-centering and crystallographically equivalent perovskite pocket volumes that originate from symmetric diammonium spacer cations. DFT-calculated charge density, band dispersion, and ELF anisotropy descriptor support the interpretation that these structural features suppress stereochemical expression and anisotropy of the Sn^2+^ lone pairs, while representative CC diagram calculations indicate that stronger lone-pair anisotropy is accompanied by larger excited-state reorganization and increased vibronic broadening of PL emission. Following such search criteria and starting from the reported DJ Pb perovskites, a new DJ Sn iodide perovskite, (4F-PDMA)­SnI_4_, is readily identified and shown to have the anticipated structural features, low ELF anisotropy, moderate excited-state reorganization, and relatively narrow and symmetrical PL emission, as well as excellent ambient stability. These results demonstrate a practical strategy to predict photophysical properties of Sn DJ perovskites from analogous Pb DJ perovskite structures. More importantly, this work demonstrates the ability to uncover key structural properties from a limited data set, encouraging the expansion of available DJ perovskite phases and exploration of data-driven and machine learning studies.^36–44^ Here, we established spacer cation molecular symmetry, equivalent perovskite pocket volumes, and suppression of metal lone-pair anisotropy as rational design criteria for designing lead-free Sn DJ perovskites toward high-performance optoelectronic applications.

## Supplementary Material





## Data Availability

The code used to calculate the ELF anisotropy descriptor and to run the SISSO++ descriptor-search and validation workflows is openly available at https://github.com/xiejiahao/elf-anisotropy-sisso.

## Notes

Calculated structural and photoluminescence parameters (XLSX)
Materials and methods, DFT and ELF analysis descriptions, crystal structure refinement data, table summarizing the CCDC structure codes for the crystal structures analyzed, additional structural parameter correlations, photographs of perovskite crystals, complete PL data with AF and FWHM analysis, perovskite pocket volume calculations, calculated band structures, one-dimensional ELF profiles, and CC diagram for select phases, absorbance data and Stokes shifts, and low-temperature PL of select DJ perovskite phases and electron–phonon coupling fittings (PDF)
