# Supplementary material for: Narrowband Emitting Tin Dion–Jacobson Perovskites through Systematic Spacer Cation Design to Suppress Metal Lone-Pair Anisotropy
Source: J Am Chem Soc. 2026 Oct 1;148(40):43289–302. doi: 10.1021/jacs.6c11969 (PMC13674271; doi:10.1021/jacs.6c11969)
Supplement: Supplementary file 1 [file ja6c11969_si_002.pdf]

Supporting Information for

Narrowband Emitting Tin Dion–Jacobson Perovskites through Systematic Spacer Cation Design to Suppress Metal Lone-Pair Anisotropy

Kristel M. Forlano[1], Jiahao Xie[2], Christopher T. Triggs[1], Jalianet Román-Matías[1], Daniel D. Kohler[1], Francesca Canestra[3], Cristina Femoni[3], David P. Lafayette[1], Ilia A. Guzei[1], Daniele Cortecchia[3], John C. Wright[1], Yanfa Yan[2], Song Jin[1]*

[1]Department of Chemistry, University of Wisconsin-Madison, Madison, Wisconsin 53706, USA

[2]Wright Centre for Photovoltaics Innovation and Commercialization, Department of Physics and Astronomy, University of Toledo, Toledo, Ohio 43606, United States

[3]Department of Industrial Chemistry Toso Montanari, University of Bologna, Bologna 40129, Italy

*Correspondence to jin@chem.wisc.edu

Experimental Methods

Chemicals and materials

All chemicals were used as received. 4-(aminoethyl)pyridine (4AEPy), 4-(aminomethyl)pyridine (4AMPy, 98%), butane-1,4-diammonium iodide ($BDAI_2$), $SnCl_2$ (98%), and H_3PO_2 (50 wt.%) were purchased from Sigma Aldrich. 3-(aminomethyl)piperidine (3AMP, 98%), and 1,5-naphthalenediamine (DAN, 98%) were purchased from TCI. 4-(aminomethyl)piperidine (4AMP, 97%) and 2-(aminoethyl)pyridine (2AEPy, 98%) were purchased from Oakwood Product Inc.. N-(2-aminoethyl)piperidine (AEP, 98%) was purchased from Across Organics. 2,2'-oxybis(ethylammonium) (OBE, 98%) and hexane-1,6-diammonium chloride ($HDA{\bullet}Cl_2$, 97%) was purchased from Ambeed. 1,4-Cyclohexane(methylamine) (1,4CDMA, 96%, mixture of isomers) was purchased from Alfa Aeser. 1,3-Cyclohexane(methylamine) (1,3CDMA, 98%, mixture of isomers) was purchased from AstaTech Inc.. PbI_2 (>98%) was purchased from Santa Cruz Biotechnology. 1,1-Dimethylbiguanide hydrochloride (DGA•HCl) was purchased from both Santa Cruz Biotechnology and Fisher Scientific Co.. (Perfluoro-1,4-phenylene)dimethanamine dihydrochloride (4F-PMDA · 2HCl, 98%) was purchased from Enamine. HI (57 wt.%) was purchased from Bean Town Chemical Cooperation.

Synthesis of lead and tin iodide perovskites

Most tin-based $(LA)SnI_4$ (LA = spacer cations) perovskites were synthesized in regular atmospheric conditions by adding the amine (LA) and $SnCl_2$ in 1:1 stoichiometric ratio to 2 mL of a 1:1 $HI:H_3PO_2$ solution to create a 0.15 M perovskite solution. $(DAN)SnI_4$ was synthesized with concentrations of 0.075 M DAN and 0.15 M $SnCl_2$. Similarly, all lead-based $(LA)PbI_4$ perovskites

were synthesized at a 0.2 M concentration of LA and PbI_2 in 2 mL of a 1:1 HI:H_3PO_2 solution with the exception of (DAN)PbI_4 at 0.1 M DAN and 0.2 M PbI_2. The solutions were heated to 140 °C on a hotplate then slowly cooled to room temperature over the course of 5 hours. Solutions with DGA were heated to only 50 °C (heating above this caused crystals to not form). With the exceptions noted below, crystals formed at the bottom of the vial upon cooling to room temperature and were removed, washed using chlorobenzene, and dried before further characterization. (DAN)SnI_4, (DAN)PbI_4, and (OBE)PbI_4 crystals were removed from solution at 100 °C to prevent formation of the spacer cation iodide salt.

Single crystal structure determination

Single-crystal X-ray diffraction was performed using a Bruker D8 VENTURE PhotonIII four-circle diffractometer with Cu Kα (λ = 1.54178 Å, operation at 50 kV and 1.1 mA) or Mo Kα (λ = 0.71073 Å, operation at 50 kV and 1.6 mA) radiation. Each structure was solved by intrinsic phasing and refined using least-squares cycles and difference Fourier maps to locate remaining non-hydrogen atoms.[1,2] All non-hydrogen atoms were refined with anisotropic displacement coefficients. All hydrogen atoms were included in the structure factor calculation at idealized positions and were allowed to ride on the neighboring atoms with relative isotropic displacement coefficients. The basic crystallographic information for the new crystal structures is summarized in Table S1.

Structural distortion parameter determination

Bond lengths, angles, various distortion parameters (Table 1 in the main text), and polyhedral volumes were calculated using VESTA software based on previously reported crystal structures (archived in the CCDC database, their CCDC codes collected in Table S2) or new crystal structures

determined in this work (CCDC codes also collected in Table S2). A polyhedron for pocket volume (Table S4) was constructed using the method described by Koegel *et. al*.[3] Metal off-centering was calculated through finding the geometrical center of the 6 iodine atoms in coordinate space and then determining the distance between the calculated center point and the coordinate of the metal atom, described by Fu *et. al.*[4]

Steady-state photoluminescence (PL) spectroscopy measurements

DJ perovskite crystals were exfoliated using the scotch tape method to expose a fresh surface. All exfoliated samples were protected from degradation by spin coating a layer of polystyrene on the surface before spectroscopic measurements. All PL spectra of n = 1 DJ perovskites were collected using a Horiba LabRAM HR Evolution confocal Raman/PL spectrometer under 405 nm (Pb) or 532 nm (Sn) CW excitation from a laser. For best accuracy when comparing PL characteristics, all PL was taken with the same collection parameters (exposure time, accumulations, laser power, etc.) within the Pb or Sn sets with the following exceptions due to maxing out the detector counts: (1,4CDMA)SnI_4, (HDA)SnI_4, (1,4CDMA)PbI_4. Low temperature PL spectra of (DGA)SnI_4 were also collected on this instrument using a Linkam THMS600 stage.

Additional low temperature PL measurements on (OBE)SnI_4 and (AEP)SnI_4 were taken using a home-built setup under 375 nm excitation with a pulsed laser diode (Horiba Delta Diode DD375L, repetition rate of 80 MHz) attenuated with a variable neutral density filter (ThorLabs NDC-100C-4M, OD ± 5%). A 50:50 plate beamsplitter both directed the excitation beam to the sample and transmitted the emitted PL. The beam was condensed through a microscope objective (Olympus UMPlanFI 10x, NA 0.30) to a ~8 µm diameter spot on the sample. Reflected PL was collected through the same objective and filtered with a 450

nm longpass filter (ThorLabs FELH0450, OD5+) before passing to an imaging spectrograph (Princeton Instruments Acton sp2300i) with a ruled grating (150 grooves/mm, 800 nm blaze) and a sCMOS camera (Andor Neo DC-152Q-FL). The sample was mounted on a modified liquid nitrogen cryostat (CRYO Industries 110-637-DND) fitted with a CaF_2 window viewport. A resistive heater controlled the temperature through a PID controller (Love Controls Series 16C). Excitation power was set to approximately 0.5 μW using the variable neutral density filter.

Diffuse reflectance spectroscopy measurements

Diffuse reflectance measurements were collected using a Jasco UV-Vis (V-570) with an integrating sphere (ISN-470). Samples were prepared by grinding single crystals onto a glass slide with carbon tape for sample adhesion. The film-like sample was then placed between the sphere sample opening and the sample holder. Spectralon was used as the 100% reflectance reference while carbon tape background was subtracted from the measured spectra. To approximate the absorption spectrum, the Kubelka-Munk transform,[5]

$$F(R) = \frac{(1-R)^2}{2R},$$

where R = R_sample/R_reference, was calculated for each sample spectrum. When the absorption cross-section is not too large, F(R) can be equated to the ratio of absorption to scattering coefficients of the objects. However, we expect the absorption to dominate for excitonic features, so our spectra may be distorted from the absorption spectrum, and peak positions will only approximate absorption peaks.

Powder X-ray diffraction (PXRD) measurements

Powder X-ray diffraction (PXRD) measurements were taken on a Bruker D8 Advance X-ray diffractometer with Cu Kα radiation on crushed single crystals of the synthesized DJ perovskites. For ambient stability monitoring of $(4FPDMA)SnI_4$, crushed single crystals were stored in dark, but ambient air conditions while relative humidity (RH) was tracked with a digital hygrometer (RH = 25-50%) during the 20-week period. Crystals were removed briefly to conduct the PXRD measurement and returned to storage conditions afterwards.

Density Functional Theory (DFT) calculations

Density functional theory calculations were performed using the Vienna Ab initio Simulation Package VASP) with projector augmented-wave pseudopotentials.[6,7] A plane-wave cutoff energy of 650 eV was employed for the PBE-level structural, electron localization function (ELF), and band-structure calculations and the Brillouin zone was sampled with a minimum k-point spacing of $0.06 \times \pi$ Å^{-1}. The exchange-correlation interactions were treated within the generalized gradient approximation using the Perdew–Burke–Ernzerhof functional,[8] complemented by Grimme's D3 semi-empirical dispersion correction for van der Waals forces.[9] Structural relaxations were conducted until the residual atomic forces were less than 0.01 eV/Å, while the lattice parameters were constrained to their experimental values. Excited-state structural optimizations were performed using constrained-occupation calculations[10] with the PBE0 hybrid functional[11] at the Γ point. The excited-state occupations were imposed directly from the ground-state optimized structures without applying any initial structural perturbation, so that the relaxed excited-state geometries reflected the structural response to the imposed electronic excitation. Configuration-coordinate diagrams[12] were constructed by linearly interpolating between the

optimized ground-state and excited-state geometries along a mass-weighted coordinate Q. Single-point total energies were calculated along this coordinate for both the ground-state and constrained excited-state electronic configurations. The resulting potential-energy surfaces were fitted near their minima using $E(q) = 1/2kq^2 + \lambda q^3$, where q is the displacement from the fitted minimum. The generalized displacement ΔQ was defined as the separation between the fitted ground-state and excited-state minima. The effective phonon energy was obtained from the harmonic force constant, and the Huang–Rhys factor was calculated as $S = E_d/(\hbar\omega_g)$, where E_d is the ground-state reorganization energy evaluated at the excited-state minimum.

Identification of symbolic-regression-based ELF anisotropy descriptor

Quantifying the stereochemical expression of the ns^2 lone pair on Sn(II) and Pb(II) remains challenging because the effect has a dual geometric–electronic character. Structural parameters such as metal off-centering displacement and bond-angle variance (σ^2) cannot reflect the electronic consequences, while basin analyses of the electron localization function (ELF) require a discrete non-nuclear attractor that frequently fails to form in condensed inorganic frameworks, where the lone-pair density merges continuously with adjacent metal–ligand bonding channels. To bridge this gap, we introduce a first-principles, site-local descriptor that captures the directional anisotropy of the electron localization along the coordination bonds, without basin partitioning.

ELF was evaluated for structures relaxed internally while retaining the experimental lattice parameters. Each SnI_6 octahedron was defined by the six nearest periodic I neighbors. One-dimensional ELF profiles were obtained by periodic tricubic interpolation of the three-dimensional ELF grid along the six Sn-I coordination directions. A lightly smoothed copy of each profile was used only to identify the first post-nuclear minimum-maximum-minimum basin; the peak position and height were then determined from the original profile by a local quadratic fit. For direction i,

d_i is the Sn-I distance, and r_i and h_i are the position and height of the selected ELF maximum, respectively (Figure S10).

The aligned d_i, r_i, and h_i values were the only primitive quantities used in the SISSO++ symbolic-regression search.[13,14] Candidate construction followed three explicit steps: combine quantities from the same Sn-I direction, summarize the six directions within each SnI_6 octahedron, and summarize inequivalent Sn sites at the material level. Table S5 lists the complete set of inputs, mathematical operations, and aggregation rules. Each direction-level expression contained at most one operation ($q_{local} = 1$). After target-independent filtering, 1,539 stable dimensionless candidates were supplied directly to SISSO++ without additional algebraic expansion.

Model discovery was evaluated by nested leave-one-material-out (LOO) cross-validation. In each of the 13 LOO cycles, one material was withheld, and candidate ranking, descriptor selection, and regression fitting were repeated using only the remaining 12 materials before predicting the withheld material. The one-descriptor (D = 1) search selected the final expression in 10 of the 13 repeated searches and the same normalized-range, maximum-site, and square structure in 11 of the 13 searches. The two-descriptor (D = 2) search did not provide a stable improvement and was not retained.

For the final expression, $M_{ij} = r_{ij}\, h_{ij}$, $A_j = [max_i(M_{ij}) - min_i(M_{ij})]/mean_i(M_{ij})$, and $A_{ELF} = max_j(A_j)$. Because A_j is the normalized range of M_{ij} over the six Sn-I coordination directions, A_{ELF} measures the relative directional variation rather than the absolute ELF magnitude and is dimensionless. $A_{ELF} = 0$ is the isotropic limit in which the six directional values are identical at every Sn site, whereas increasing A_{ELF} indicates progressively stronger directional asymmetry in the Sn-centered ELF. Taking the maximum over inequivalent Sn sites retains the most anisotropic local Sn environment in each structure. Thus, A_{ELF} provides a static electronic measure of the directional

stereochemical expression of the Sn(II) $5s^2$ lone pair. The complete nested search gave a leave-one-out cross-validated coefficient of determination Q^2_{LOO} = 0.731, a root-mean-square error (RMSE) of 67.7 meV, and a mean absolute error (MAE) of 47.7 meV. After the analytical form was fixed, leave-one-out refitting of only the linear regression coefficients gave Q^2_{LOO} = 0.877 and RMSE = 45.8 meV. Across all 13 Sn DJ perovskites, A_{ELF}^2 correlates strongly with the measured PL FWHM (R^2 = 0.907; Figure 4d in the main text). Its relationships with structural distortion in the experimental and internally relaxed structures are shown in Figure S12. Within the present library of 14 Sn DJ perovskites (when the newly identified (4FPDMA)SnI_4 is also included), natural gaps in the descriptor distribution suggest useful descriptive ranges: $A_{ELF}^2 < 0.001$, $0.001 \leqslant A_{ELF}^2 < 0.03$, and $A_{ELF}^2 \geqslant 0.03$, corresponding to weak, moderate, and strong static ELF anisotropy, respectively. These divisions are equivalent to $A_{ELF} < 0.032$, $0.032 \leqslant A_{ELF} < 0.173$, and $A_{ELF} \geqslant 0.173$.

Supplemental Figures and Tables

Table S1. Crystallographic information for the new crystal structures of *n* = 1 Dion-Jacobson phase tin perovskites reported herein.

Compound	$(DGA)SnI_4$	$(1,3CDMA)SnI_4$	$(1,4CDMA)SnI_4$	$(HDA)SnI_4$	$(4FPDMA)SnI_4$
Crystal system	monoclinic	orthorhombic	monoclinic	monoclinic	monoclinic
Space group	$P2_1/c$	*Pnma*	$P2_1/c$	$P2_1/c$	$P2_1/c$
a/Å	12.6626(13)	8.955(6)	12.249(3)	11.7021(6)	12.997(3)
b/Å	13.0065(12)	24.923(11)	8.7562(19)	8.3809(4)	8.5375(13)
c/Å	20.254(2)	8.323(5)	8.6910(13)	8.9574(5)	8.486(3)
β/°	98.136(7)	90	95.728(12)	107.6860(10)	97.600(13)
Volume/$Å^3$	3302.2(6)	1857.4(19)	927.5(4)	836.97(8)	933.4(4)
Z	8	4	2	2	2
ρ_{calc}g/cm^3	3.047	2.756	2.759	2.954	2.976
Reflections collected	31255	31235	40368	7883	37003
Independent reflections	6697 [R_{int} = 0.0451, R_{sigma} = 0.0461]	2904 [R_{int} = 0.0428, R_{sigma} = 0.0201]	2841 [R_{int} = 0.0449, R_{sigma} = 0.0192]	1471 [R_{int} = 0.0358, R_{sigma} = 0.0264]	3765 [R_{int} = 0.0491, R_{sigma} = 0.0261]
Data/restraints/parameters	6697/3/277	2904/0/74	2841/0/71	1471/0/62	3765/0/89
Goodness-of-fit on F^2	1.127	1.028	1.026	1.302	1.095
Final R indexes [I>=2σ (I)]	R_1 = 0.0359, wR_2 = 0.1023	R_1 = 0.0231, wR_2 = 0.0508	R_1 = 0.0253, wR_2 = 0.0599	R_1 = 0.0194, wR_2 = 0.0457	R_1 = 0.0290, wR_2 = 0.0657
Final R indexes [all data]	R_1 = 0.0421, wR_2 = 0.1062	R_1 = 0.0293, wR_2 = 0.0539	R_1 = 0.0335, wR_2 = 0.0634	R_1 = 0.0199, wR_2 = 0.0459	R_1 = 0.0343, wR_2 = 0.0694
Largest diff. peak/hole / e $Å^{-3}$	0.95/-0.94	1.03/-0.93	1.07/-0.57	0.79/-0.72	0.59/-1.70

Table S2. CCDC codes for structures analyzed in Table 1.

Spacer	Sn structure code	Pb structure code
1,3CDMA	2557042 (this work)	2267531
1,4CDMA	2557040 (this work)	2357879
HDA	2557071 (this work)	1420433
DGA	2557039 (this work)	2173697
3AMP	2202681	1831521
4AMP	2322607	1831525
BDA	2303744	853207
2AEPy	1949807	1944786
4AEPy	1949809	1944782
4AMPy	2157414	1942547
AEP	657420	1841683
OBE	2204975	2306554
DAN	853212	2327775
4F-PDMA	2557041 (this work)	2069502

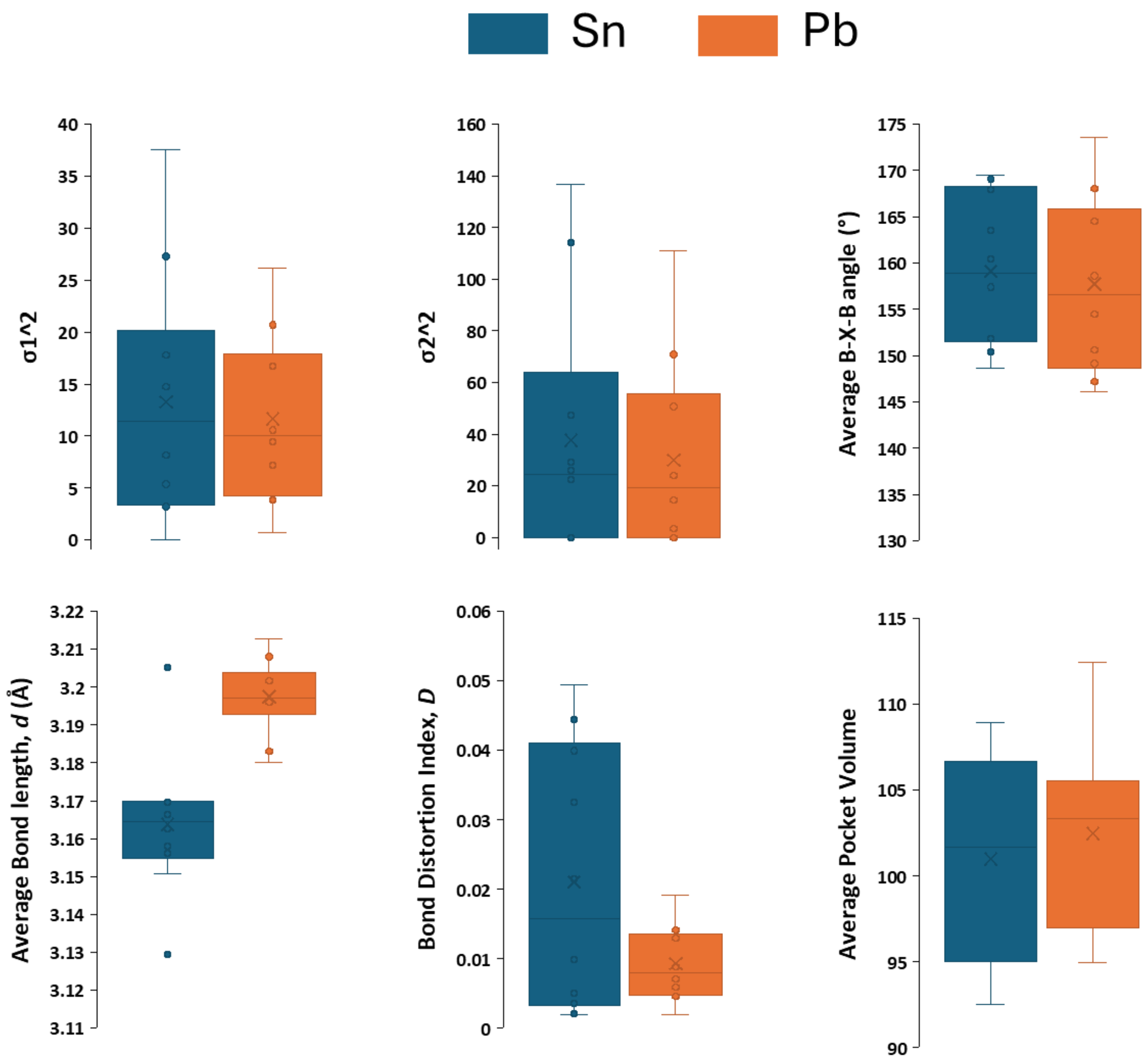

Figure S1. Box and whiskers plots comparing the ranges of various calculated structural parameters calculated for the Sn (blue) versus Pb (orange) DJ iodide perovskites.

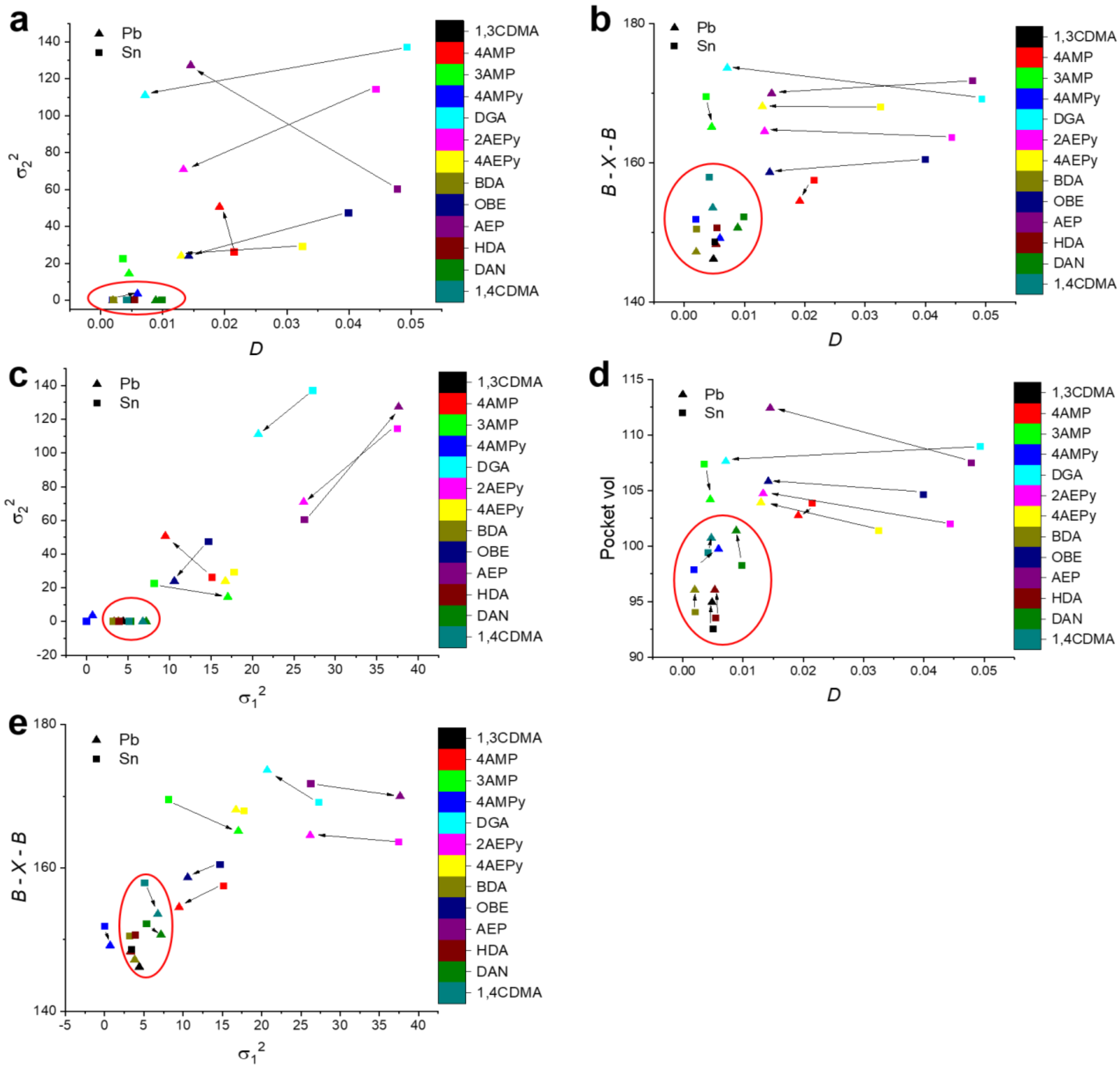

Figure S2. Examples of other correlation plots between various structural parameters that also reveal clustered group of DJ Pb/Sn iodide perovskite structures in the library of 13 pairs of DJ Pb/Sn perovskites, marked by red circles. a) D vs σ_2^2, b) D vs B-X-B angle, c) σ_1^2 vs σ_2^2, d) D vs pocket volume, and e) σ_1^2 vs B-X-B angle. The arrows connect the pairs of DJ Sn (square) to Pb (triangle) iodide perovskites sharing the same spacer cation, which is color coded according to the legends on the right.

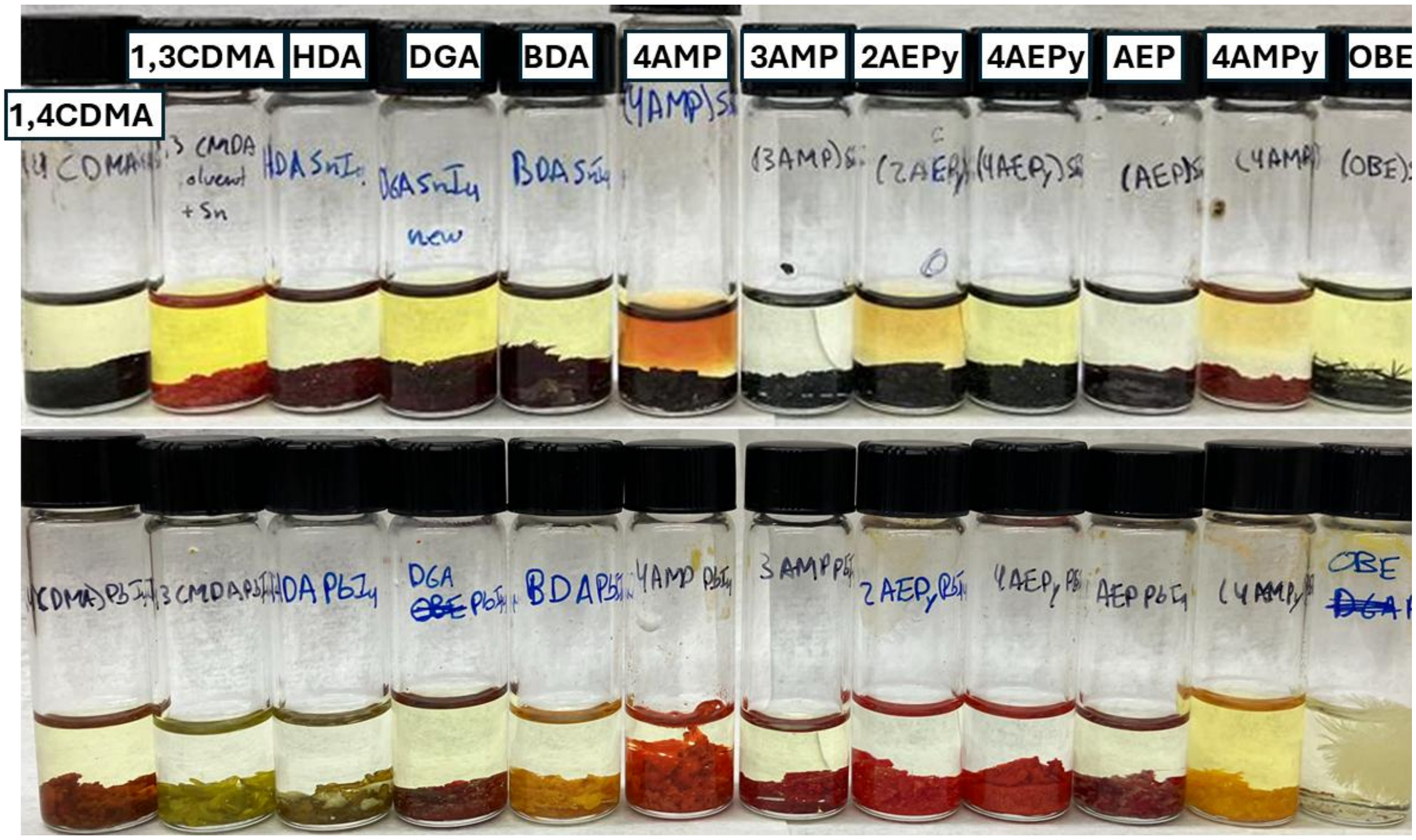

Figure S3. Photographs of synthesized crystals of Sn (top) and Pb (bottom) iodide DJ perovskites, as grown in the mother liquors. Vials are lined up top-to-bottom between the Sn and Pb phases with the same spacer cation, including vials of alternate Pb products made using the spacer cations of BDA, HDA, 4AMPy, and OBE (for which there were complications in the synthesis of these targeted DJ Pb iodide perovskites, as discussed in the main text and methods). Not pictured: $(DAN)BI_4$.

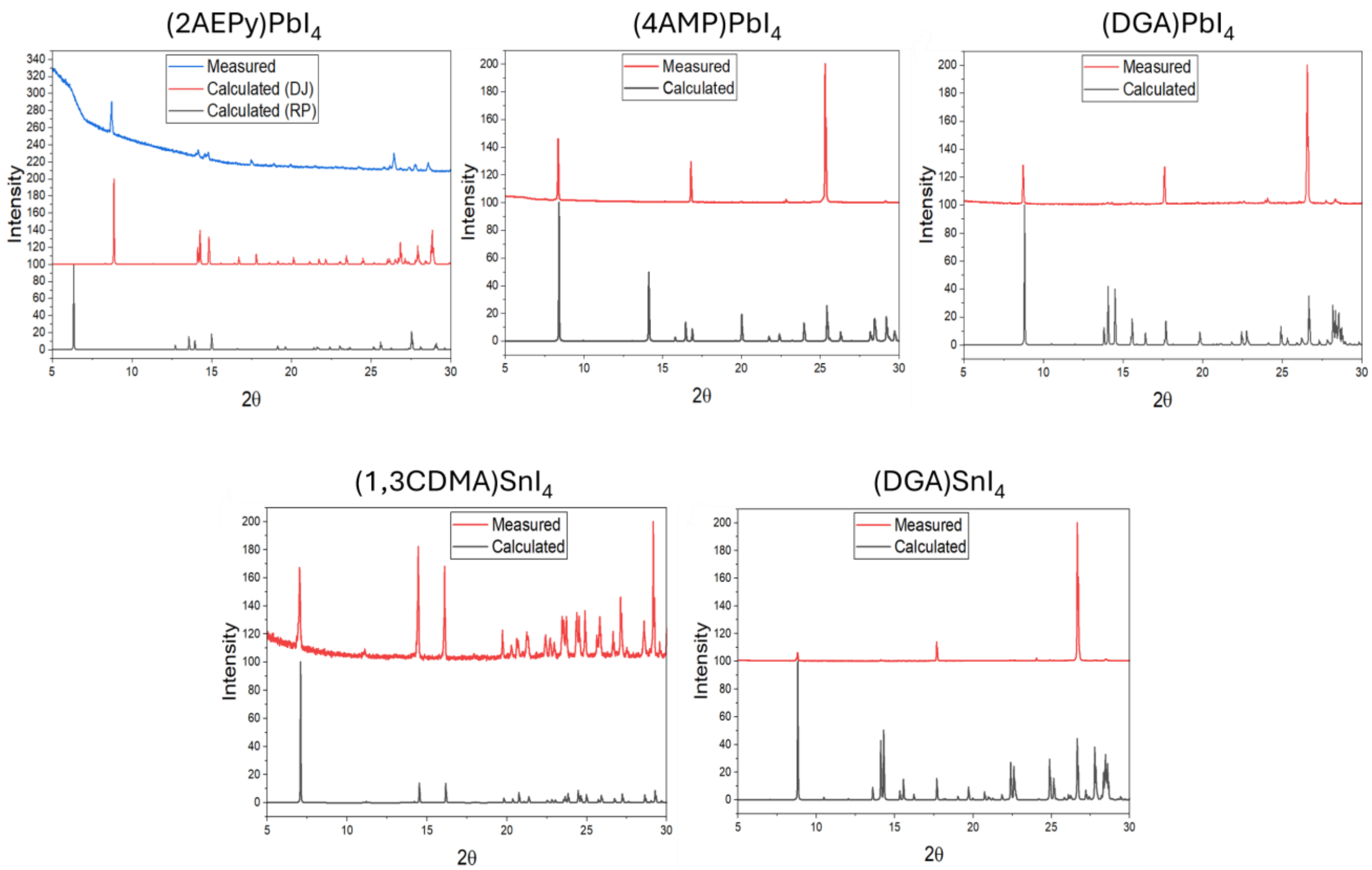

Figure S4. PXRD patterns of select DJ perovskite phases comparing measured vs calculated patterns, confirming successful synthesis of the expected 2D perovskite compounds. The 2AEPy spacer can form both a RP and DJ Pb perovskite structure, so the calculated patterns for both are displayed. Measured PXRD patterns show significant preferred orientation, as is common in 2D perovskites. Some predicted PXRD peaks might not be observed due to preferred orientation, however the experimental PXRD patterns do not show any diffraction peaks that are not predicted from the single crystal structures, therefore confirming that there are no impurity phases.

Table S3. Number of exfoliated objects measured using PL spectroscopy for each phase.

Spacer cation	Sn - Number of objects	Pb – Number of objects
1,3CDMA	6	6
4AMP	10	4**
3AMP	10	6
4AMPy	11	0*
DGA	10	10
2AEPy	10	4**
4AEPy	12	6
BDA	10	0*
OBE	10	3**
AEP	9	9
HDA	10	0*
DAN	11	9
1,4CDMA	10	11

*Phases could not be synthesized herein.

**Phase prone to large defect emission so limited plates with high excitonic recombination emission were found.

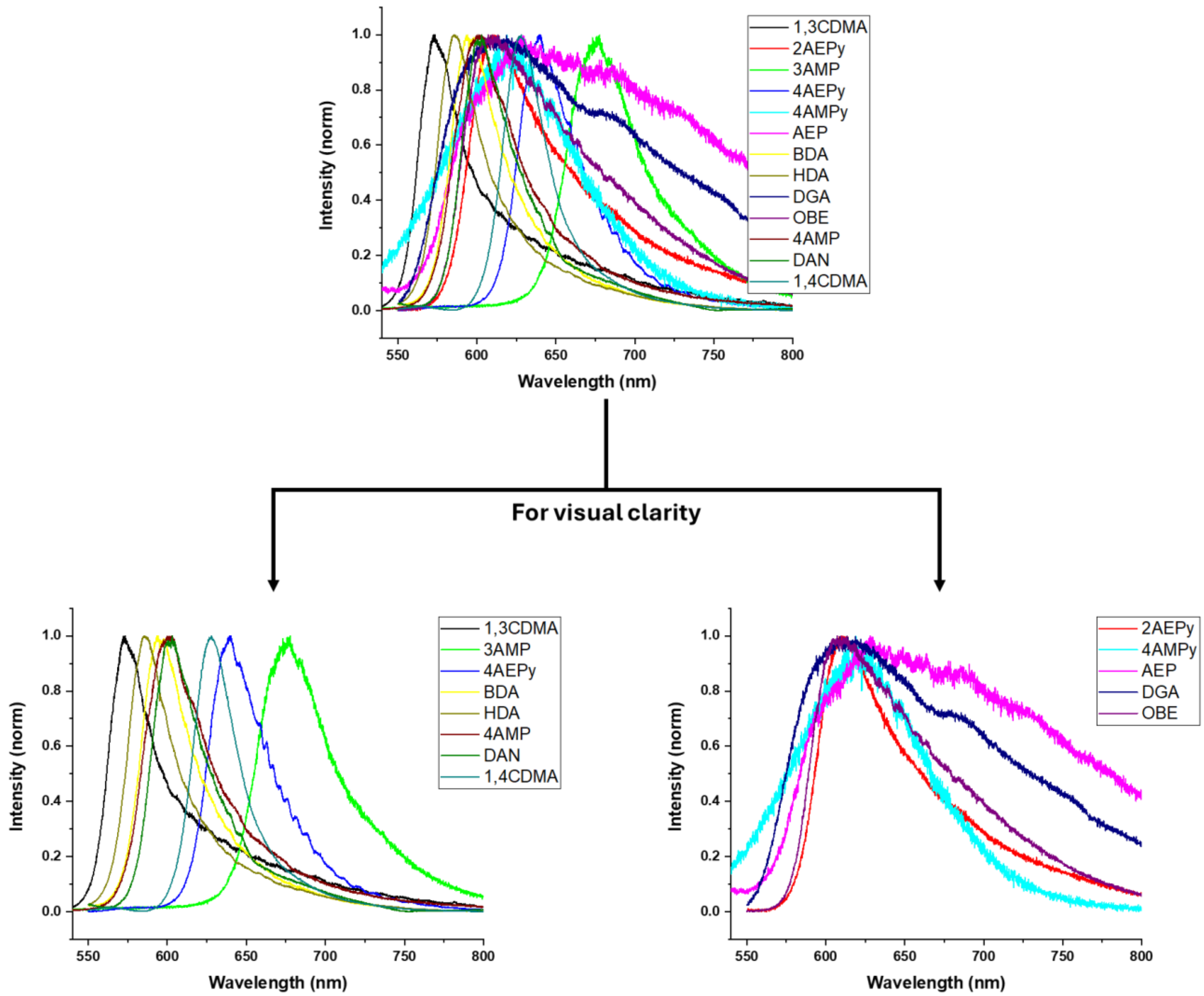

Figure S5. Representative PL spectra for all 13synthesized Sn iodide DJ perovskites. For visual clarity, due to the number of traces spreading over a large wavelength range, we plot the "narrow band" and "broad band" emitters separately at the bottom. This is a qualitative, arbitrary separation.

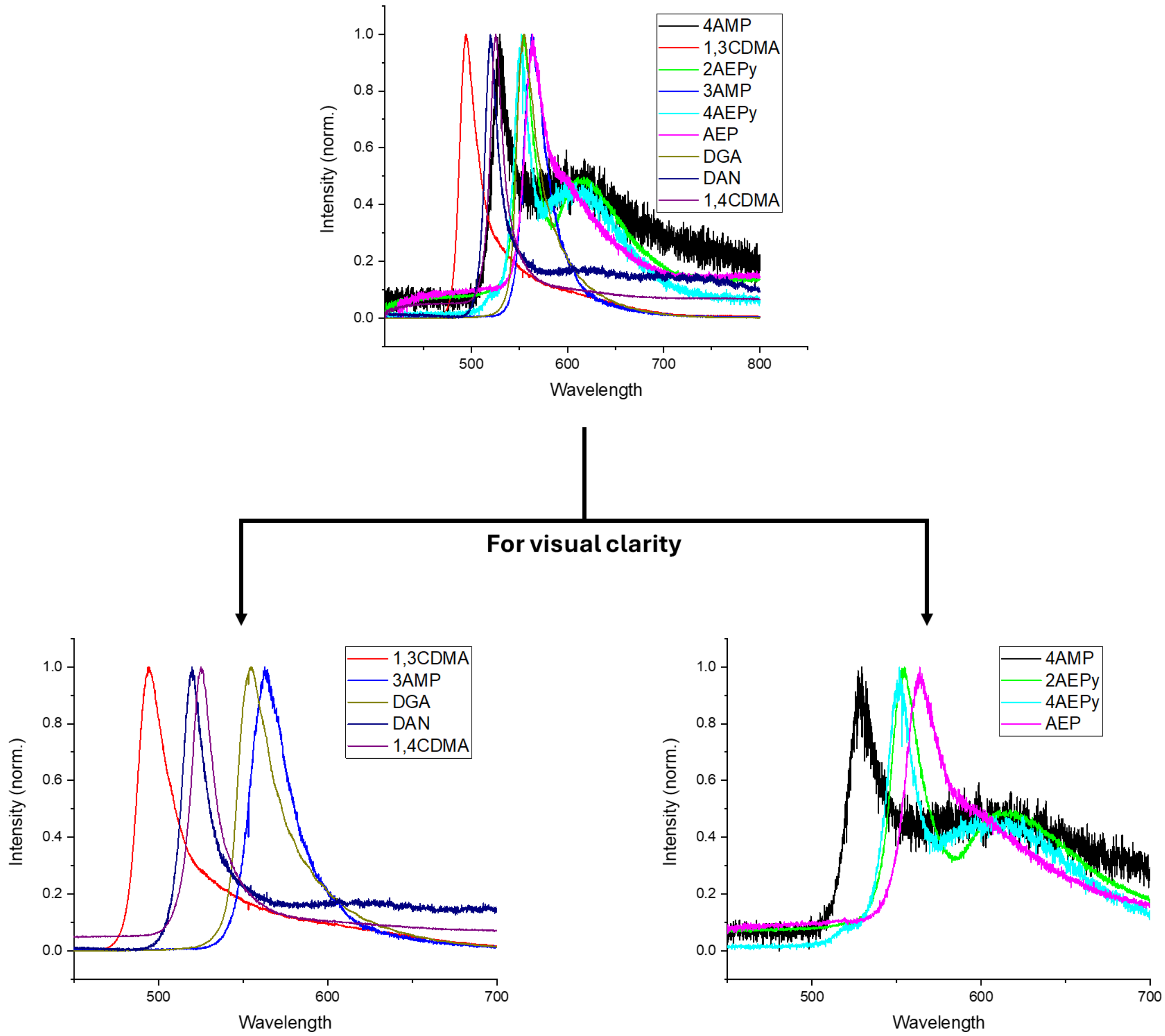

Figure S6. Representative PL spectra for all synthesized Pb iodide DJ perovskites. For visual clarity, due to number of traces, we plot the phases with persistent second emission peaks separately at the bottom. This is a qualitative, arbitrary separation. We note that some of the phases plotted in the bottom lefthand graph also exhibit a minor broad, red-shifted emission band sometimes, but PL spectra without such emission could be found. Phases in the righthand graph always exhibit another strong and broad red-shifted emission band, which might be attributed to defect emission.

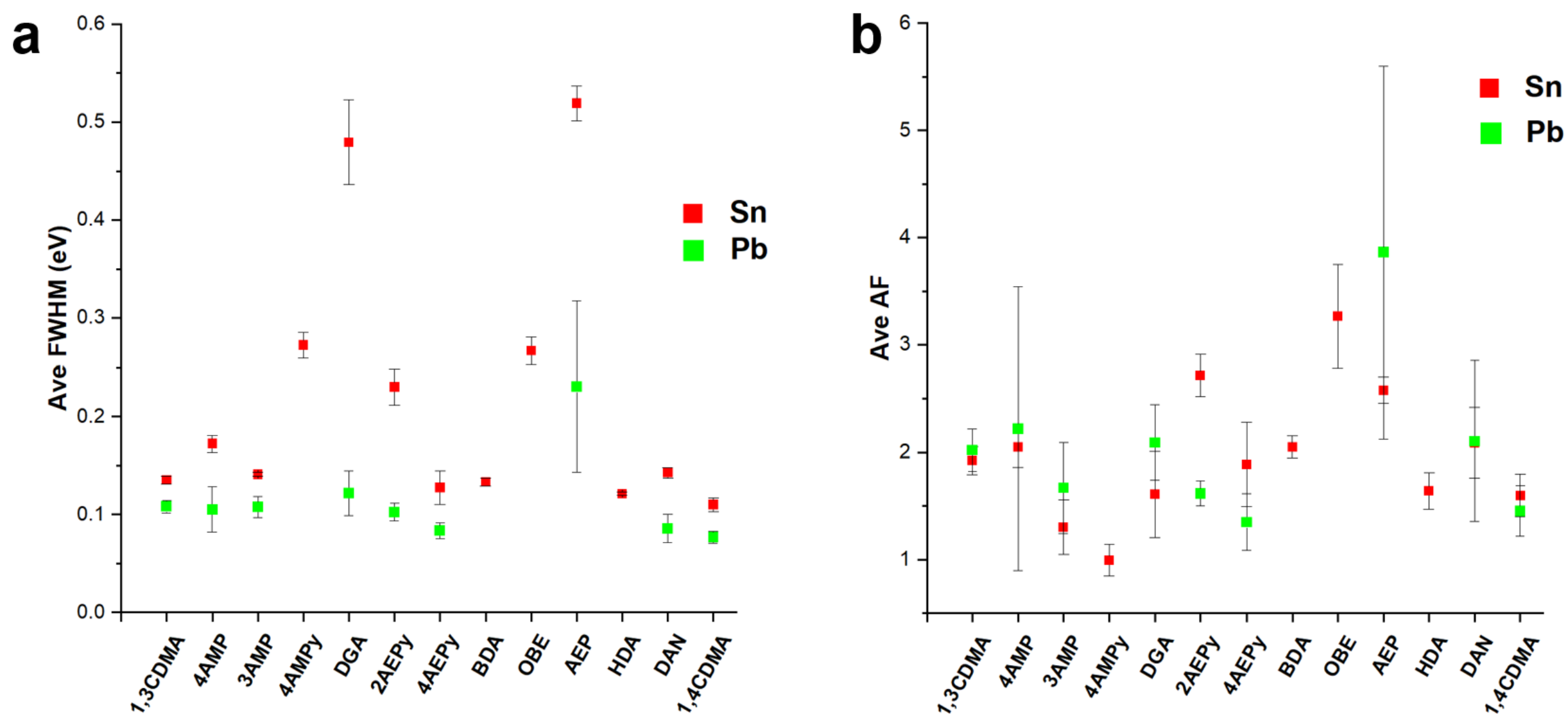

Figure S7. a) FWHM and b) AF calculated from the PL emission peaks for each Sn (red) and Pb (green) DJ iodide perovskite phase. Error bars represent standard deviations.

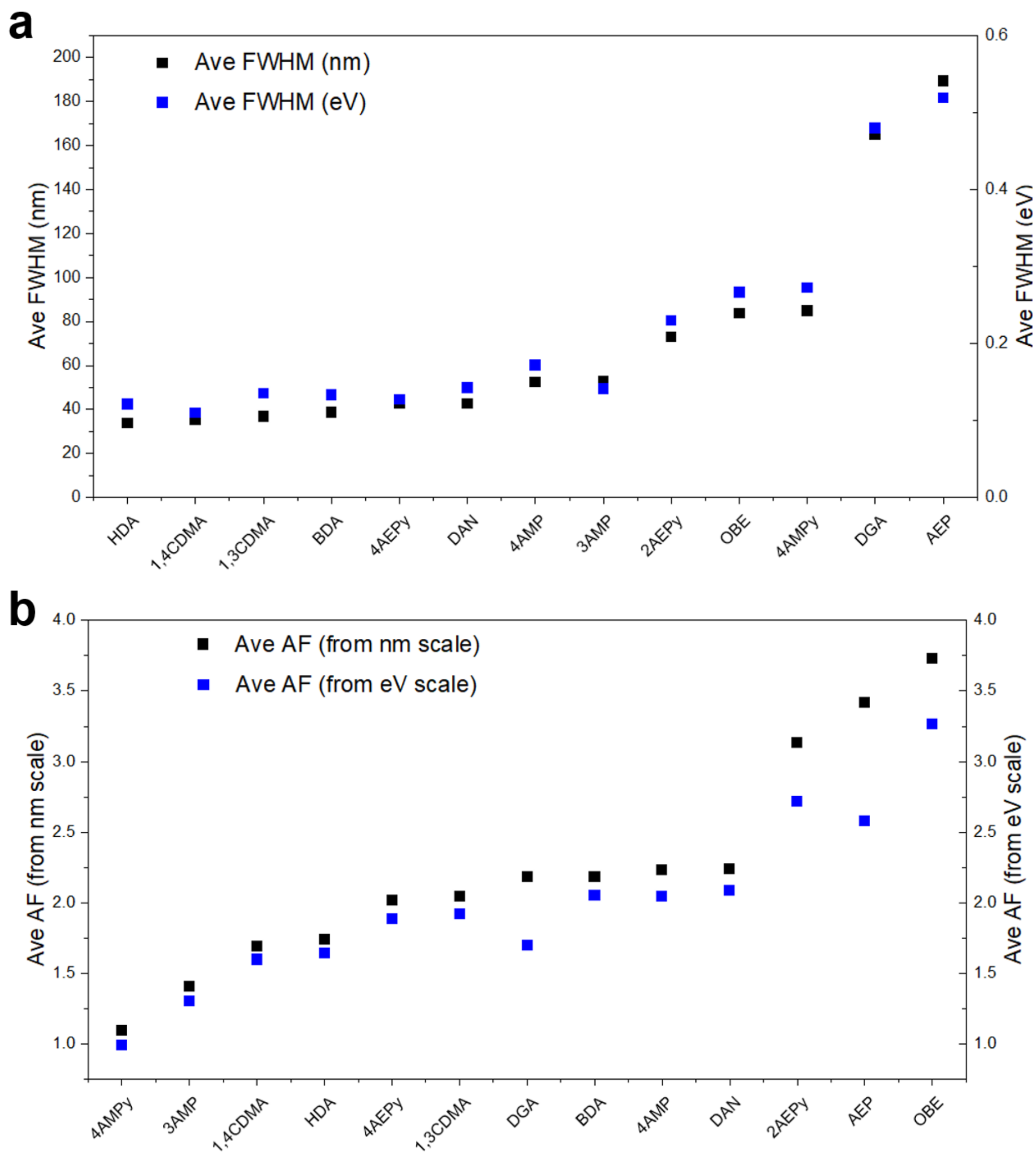

Figure S8. Comparison of a) FWHM and b) AF calculated from the PL spectra of Sn DJ perovskite in wavelength vs eV scales. FWHM trends closely between both scales. AF overall keeps to a similar trend, but deviates more substantially in DJ Sn perovskites with large, broad PL tails (LA = OBE, AEP, 2AEPy, DGA).

Table S4. Pocket volumes for 8 pairs of DJ Pb/Sn iodide perovskites with asymmetric spacer cations.

		1. Sn (Å^3)	2. Sn (Å^3)	ΔSn (Å^3)	1. Pb (Å^3)	2. Pb (Å^3)	ΔPb (Å^3)
4AMP	Ring vs chain	102.5454	105.164	2.6186	2 types of ring/chain pockets (4 pocket volumes total)		
3AMP	Ring vs chain	108.7896	105.9438	2.8458	97.2275	111.1768	13.9493
4AMPy	Ring vs chain	2 types of ring/chain pockets (4 pocket volumes total)			99.0972	100.3805	1.2883
DGA	N end vs C end	113.0982	104.8203	8.2779	105.5784	109.6767	4.0983
2AEPy	Ring vs chain	98.6917	105.2471	6.5554	101.8332	107.6528	5.8196
4AEPy	Ring vs chain	99.5343	103.2395	3.7052	102.7658	105.1007	2.3349
OBE	Symmetric molecule, but two different orientations into pocket	104.8261	104.4424	0.3837	105.8811	105.7768	0.1043
AEP	Ring vs chain	102.6174	112.352	9.7346	110.6128	114.2574	3.6446

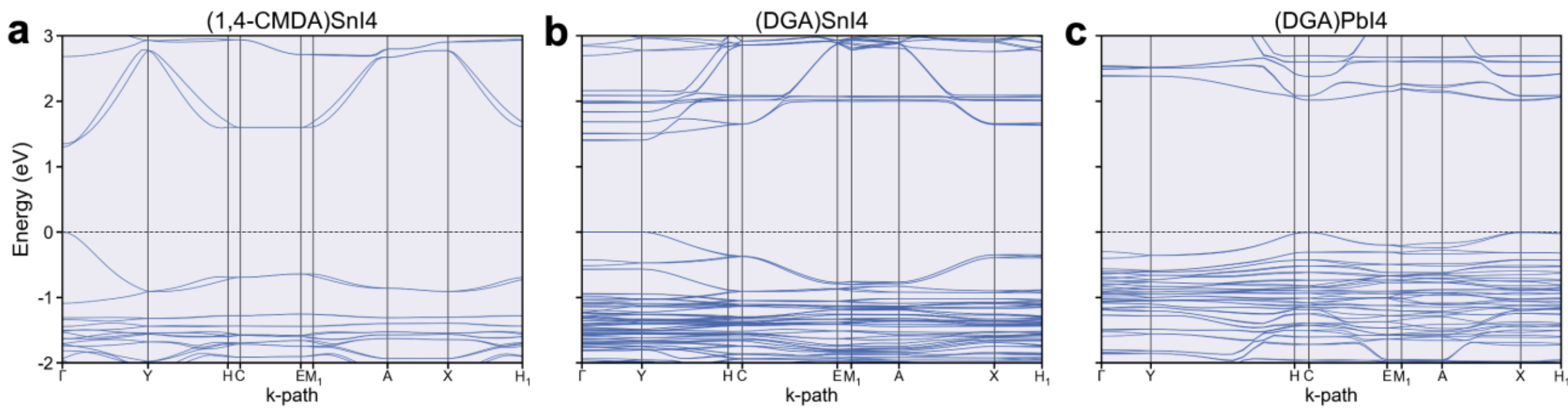

Figure S9. Band structures for (1,4CDMA)SnI_4 (a), (DGA)SnI_4 (b), and (DGA)PbI_4 (c).

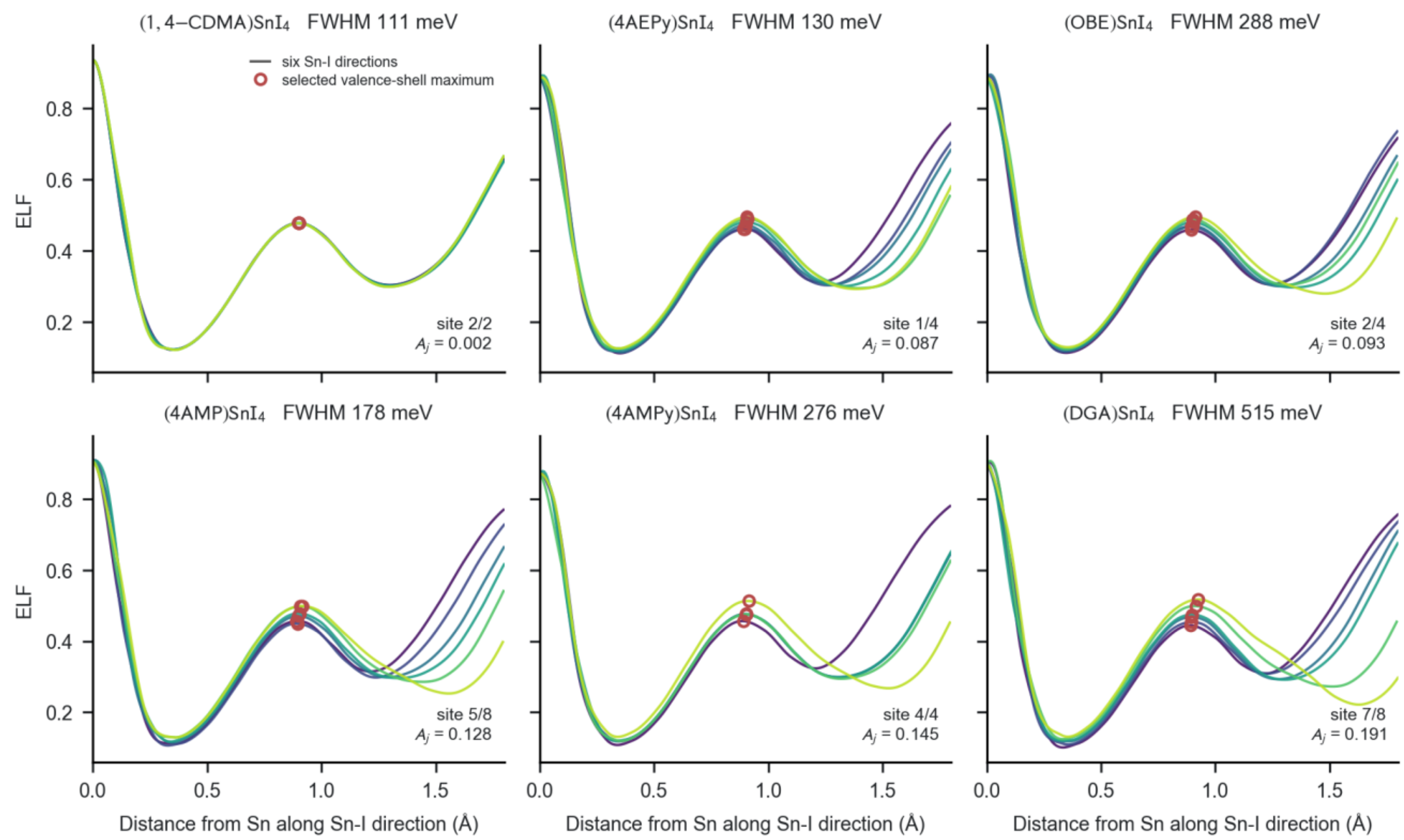

Figure S10. Identification of the first Sn-centered valence-shell ELF maximum along the six Sn-I coordination directions using the examples of 6 LASnI_4 phases. Representative sites span the ELF-anisotropy range of the material series. Open circles mark maxima selected from the first post-nuclear minimum-maximum-minimum basin and refined on the unsmoothed profile by a local quadratic fit. The low-anisotropy (1,4CDMA)SnI_4 site shows nearly overlapping profiles, whereas the high-anisotropy (DGA)SnI_4 site shows distinct first-maximum regions and a larger spread of directional ELF descriptors.

Table S5. Primitive quantities, mathematical operations, and aggregation rules used to generate the SISSO++ candidate space.

Search level	Allowed input or operation	Notes
Primitive quantities	d_i; r_i; h_i	Direction-level physical inputs.
Direction-level operations	x; $1/x$; x^2; square root; +; -; absolute difference; multiplication; division	Direction-level candidate expressions constructed from aligned inputs.
Within one SnI_6 octahedron	mean; standard deviation (SD); coefficient of variation (CV); normalized range; MAD; IQR; minimum; maximum; two-largest-deviation mean	One site-level scalar for each SnI_6 octahedron.
Across inequivalent Sn sites	mean; median; maximum; range; CV	One material-level scalar for each candidate expression.
Final transform	x; $1/x$; x^2; square root	Final material-level candidate descriptor.
Candidate screening	finite; nonconstant; dimensionally valid; dimensionless; numerically stable; nonredundant	Retained SISSO++ input feature space (1,539 candidates).

Note: CV = SD/|mean|; normalized range = (maximum - minimum)/|mean|; MAD is the median absolute deviation; IQR is the interquartile range; and the two-largest-deviation mean is the mean of the two largest absolute deviations from the site mean.

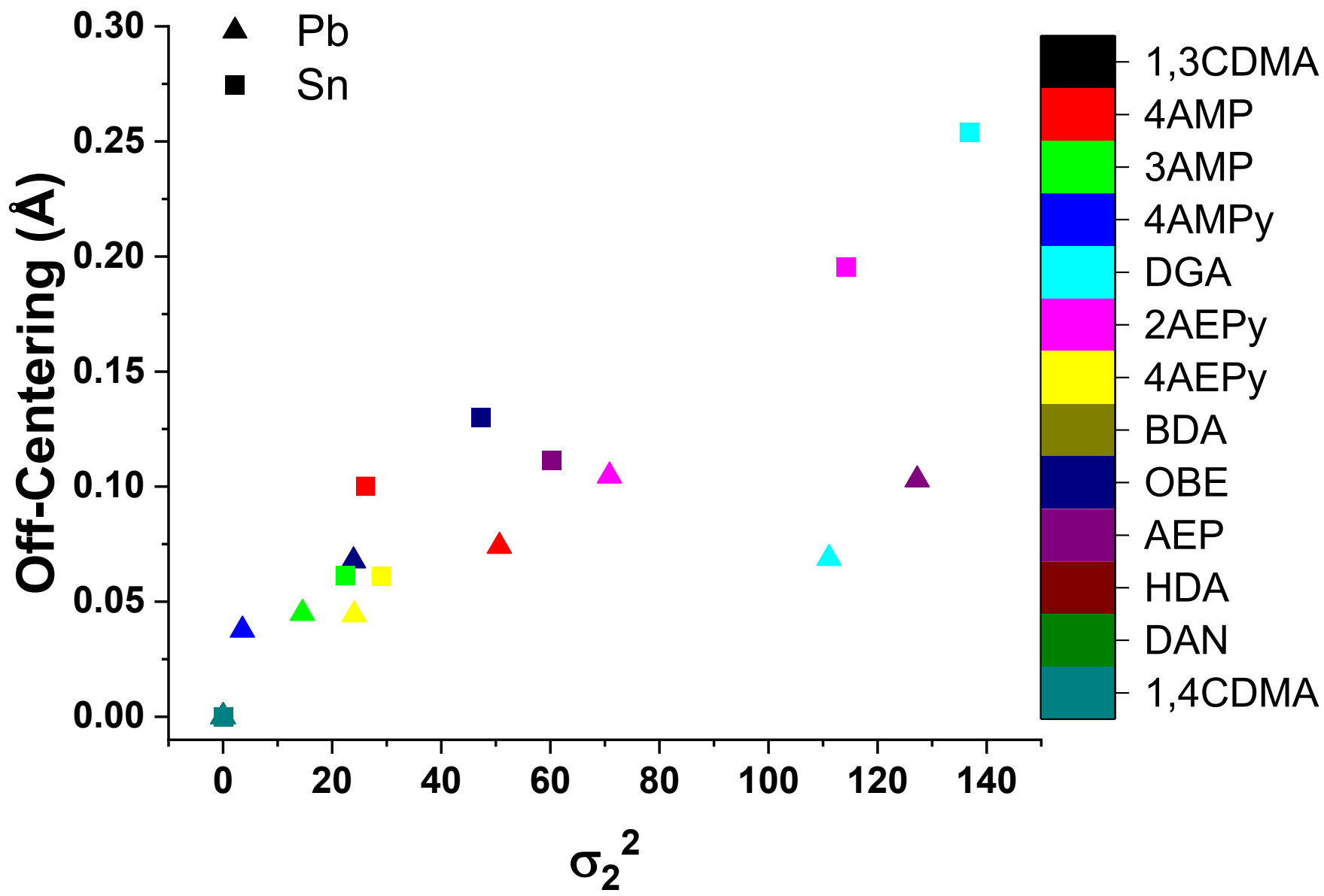

Figure S11. Correlation plot between σ_2^2 and metal off-centering for the library of 13 pairs of Pb/Sn iodide DJ perovskites. All 5 pairs of clustered DJ perovskite structures ((BDA)BI_4, (HDA)BI_4, (DAN)BI_4, (1,3CDMA)BI_4, and (1,4CDMA)BI_4) and (4AMPy)SnI_4 overlap at (0,0) due to their zero metal off-centering and σ_2^2 parameters.

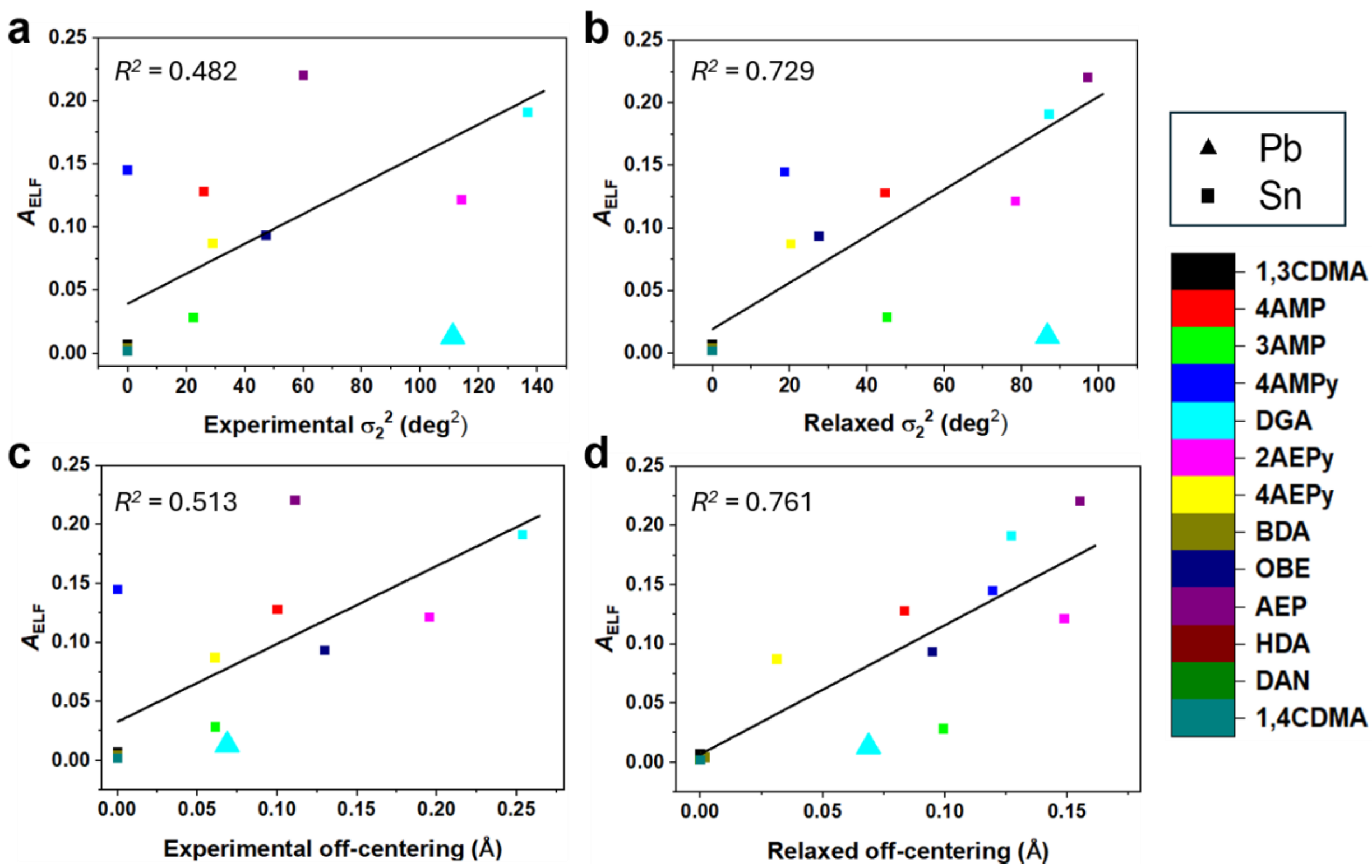

Figure S12. Relationship between relaxed-structure ELF anisotropy descriptor (A_{ELF}) and structural distortion evaluated at two structural levels for calculated DJ Sn iodide perovskite structures with added (DGA)PbI_4 structure (triangle). A_{ELF} is compared with (a) σ_2^2 from experimental crystal structures, (b) σ_2^2 from internally relaxed structures, (c) metal off-centering from experimental structures, and (d) metal off-centering from internally relaxed structures. Markers for (DGA)PbI_4 structure are enlarged for visibility to highlight the difference in calculated anisotropy of the ELF (A_{ELF}) despite high σ_2^2. In contrast, the correlation between the calculated anisotropy of the ELF (A_{ELF}) and metal off-centering is much better.

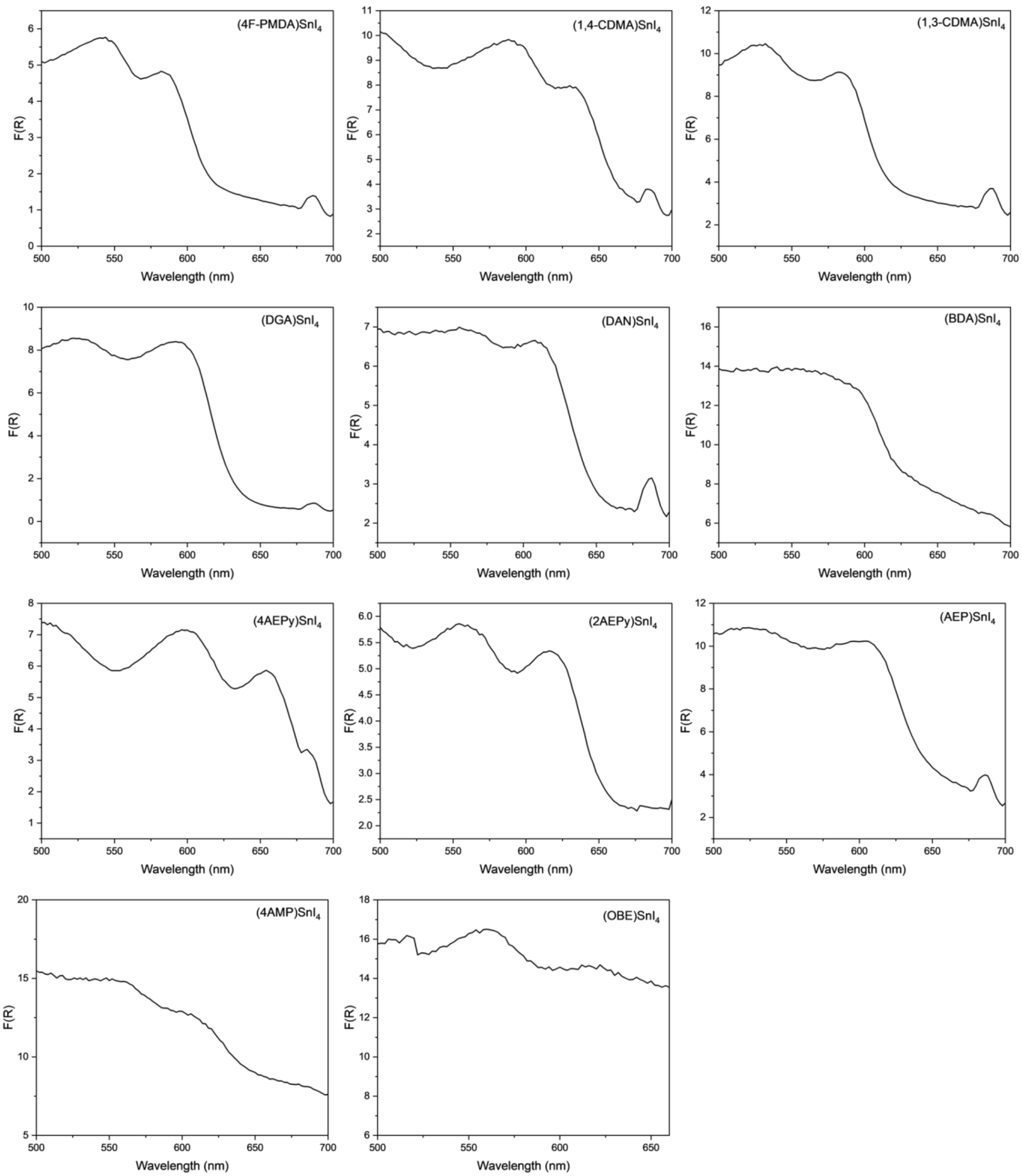

Figure S13. Diffuse reflectance spectra for most (LA)SnI_4 compounds, excluding (HDA)SnI_4, (3AMP)SnI_4, and (4AMPy)SnI_4 due to poor resolution. The small peak at ~680 nm is an instrument artifact.

Table S6. Stokes shifts of DJ Sn iodide perovskite phases $LASnI_4$. We note that the absorbance excitonic peak position is determined from diffuse reflectance spectra, which should exhibit similar resonances but some uncertainty in position (up to the width of the feature) is expected, leading to "negative" Stokes shifts. See Methods section for further discussion.

LA	Ave. PL center (nm)	Approx. Abs excitonic peak (nm)	Approx. Stokes shift (nm)
4F-PDMA	596.2 +/- 2.5	586	10
1,4CDMA	627.97 +/- 0.39	634	-6
1,3CDMA	575.5 +/- 3.1	586	-10
DGA	630.5 +/- 9.2	596	34
DAN	601.3 +/- 1.9	610	-9
BDA	593.53 +/- 0.45	590	4
4AEPy	639.71 +/- 0.47	603	36
2AEPy	610.3 +/- 1.9	618	-8
AEP	627.2 +/- 1.3	606	21
4AMP	604.62 +/- 0.78	610	-5
OBE	601.2 +/- 8.1	562	39

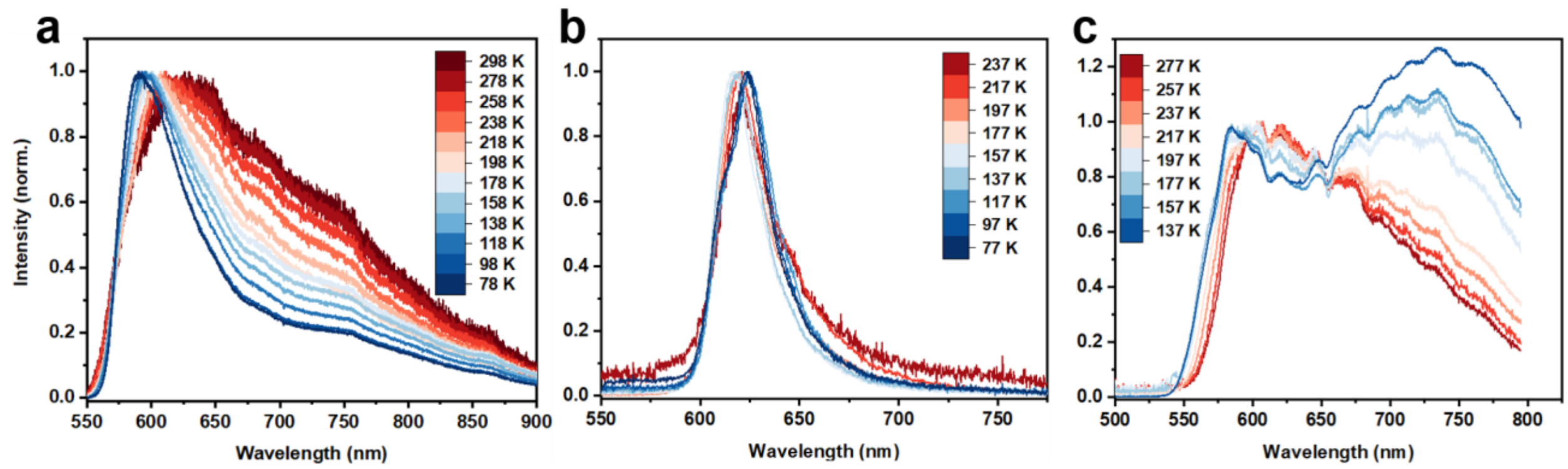

Figure S14. Low temperature PL spectra of a) $(DGA)SnI_4$, b) $(OBE)SnI_4$, and c) $(AEP)SnI_4$. All spectra are normalized; $(AEP)SnI_4$ is normalized to the intensity of the excitonic peak (~550 – 625 nm).

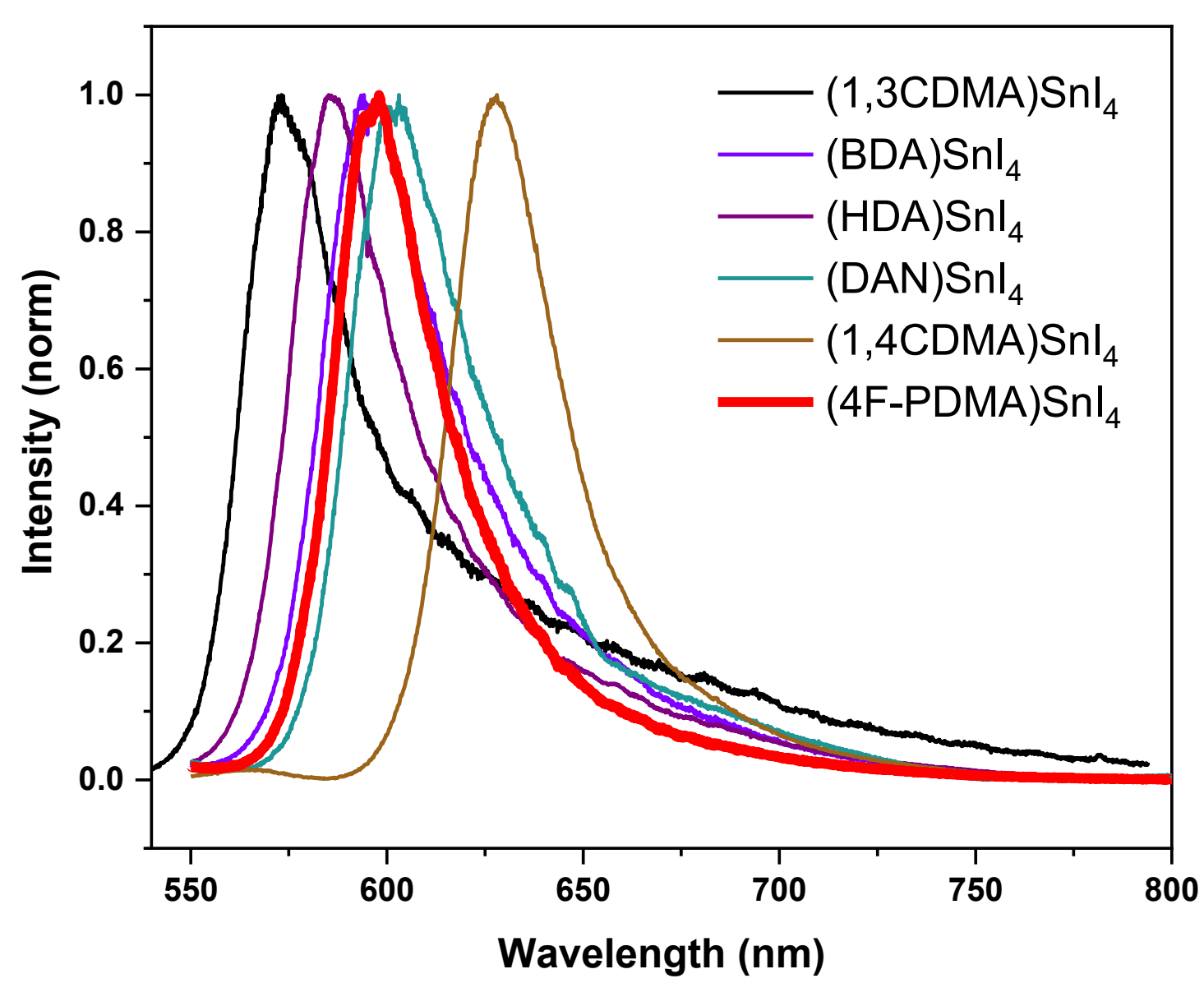

Figure S15. PL spectra of all Sn iodide DJ perovskites that fall into the clustered region and exhibit narrow and symmetric PL peaks. The PL spectrum of the new $(4F\text{-}PDMA)SnI_4$ that matches well with the other PL emissions.

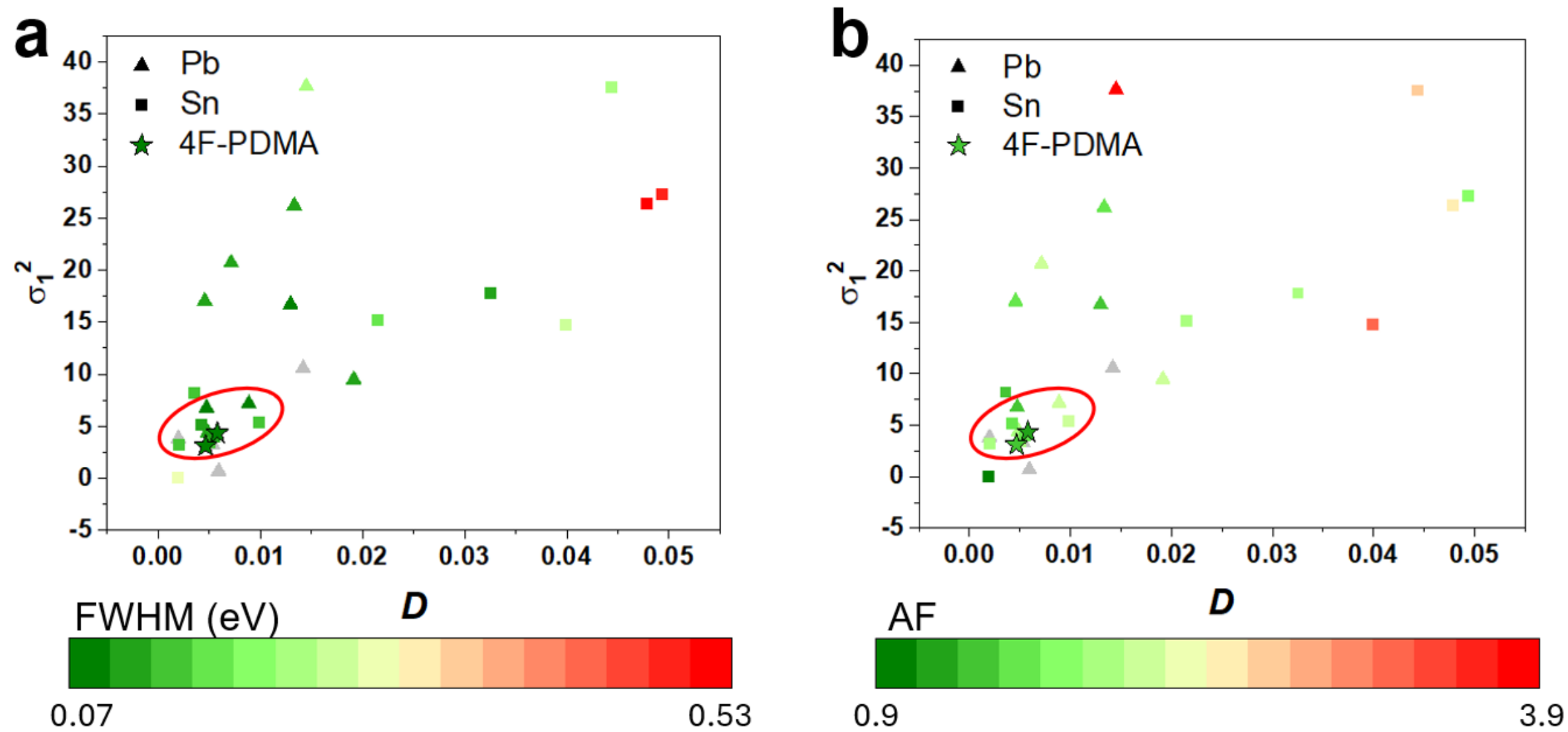

Figure S16. Correlation plots of a) FWHM and b) AF parameters of the PL emission (based on the color scales at bottom, calculated in eV scale) with the structural parameters of *D* vs σ_1^2. Dark green stars for 4F-PDMA phases indicate narrow FWHM and small AF. Grey symbols indicate no experimentally determined PL properties due to synthetic difficulties. These 2D plots present essentially the same information as the 3D plots in Figure 3c, 3d of the main text, the only difference is that the data points for (4F-PDMA)PbI_4 and (4F-PDMA)SnI_4 are added herein.

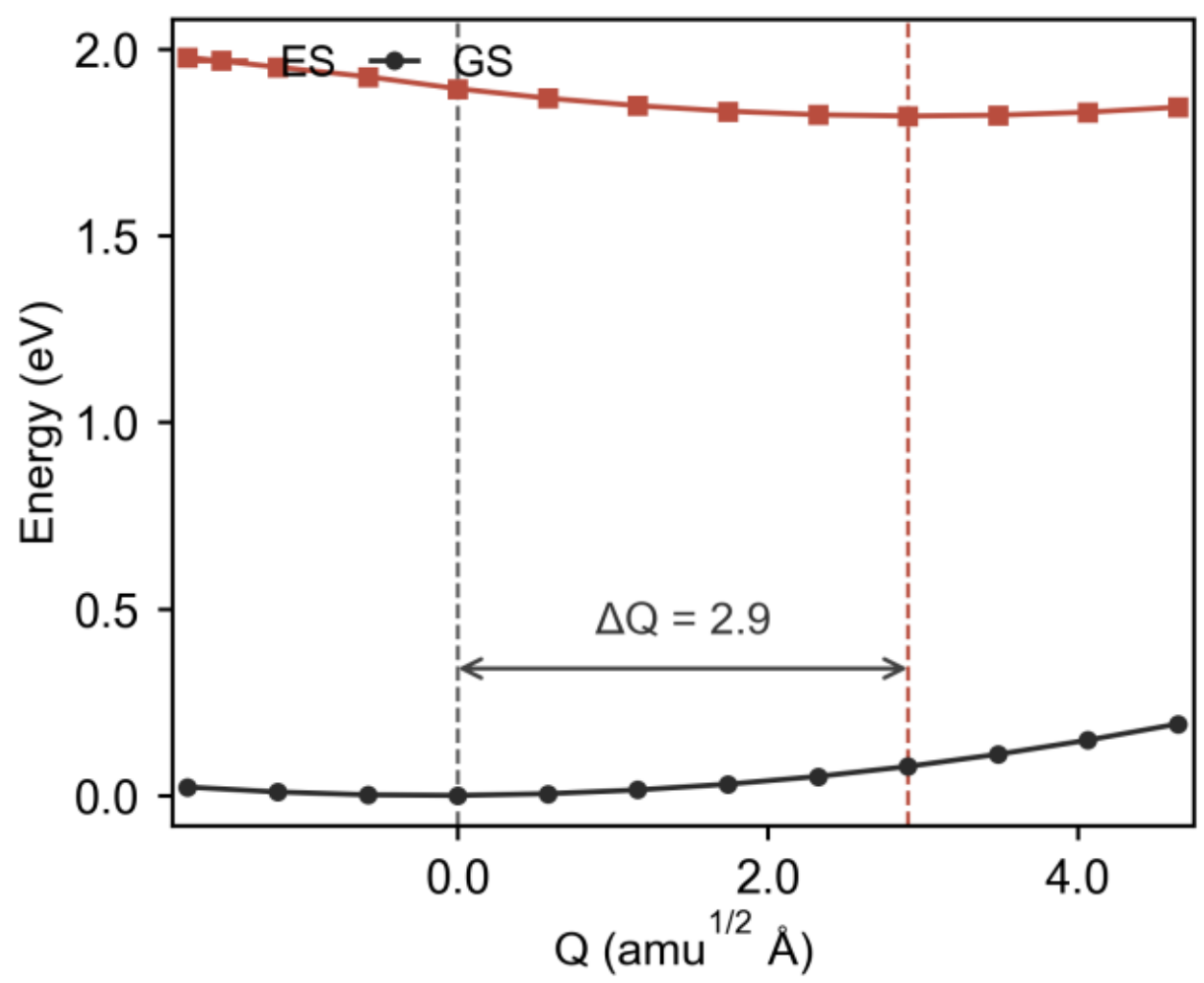

Figure S17. Configuration-coordinate diagram for (4F-PDMA)SnI_4, showing an intermediate excited-state structural reorganization between the narrow-band emitting (1,4CDMA)SnI_4 and broad-band emitting (DGA)SnI_4 references. The Huang–Rhys factor is 8.97 and the generalized displacement is 2.90 $amu^{1/2}$ Å.

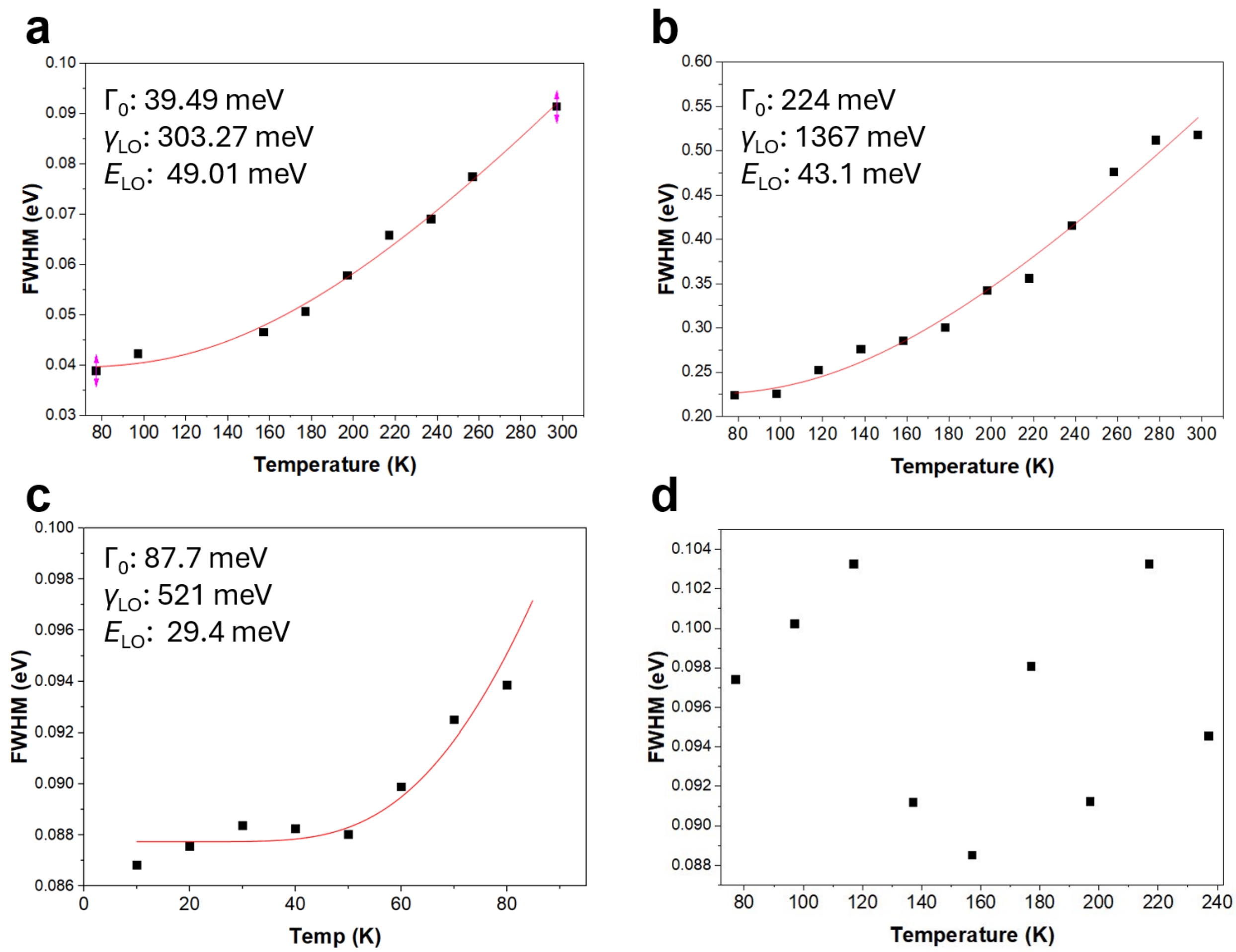

Figure S18. Plots of temperature vs FWHM for a) (4F-PDMA)SnI_4, b) (DGA)SnI_4, c) (3AMP)SnI_4, and d) (OBE)SnI_4. Data are fit according to the equation $\Gamma(T) = \Gamma_0 + \Gamma_{LO} = \Gamma_0 + \gamma_{LO}/(e^{E_{LO}/k_BT}-1)$, but the data for (OBE)$SnI_4$ did not yield a reliable fitting. Data for c) (3AMP)SnI_4 were reported in ref. 17 and provided by the author, Prof. Anita Ho-Baillie of U. of Sydney.

Table S7. Collection of electron-phonon coupling fitting values for the equation $\Gamma(T) = \Gamma_0 + \Gamma_{LO} = \Gamma_0 + \gamma_{LO}/(e^{E_{LO}/k_BT}\text{-}1)$ of the various DJ Pb and Sn iodide perovskites reported or fitted here.

LA	Pb/Sn	Γ_0 (meV)	γ_{LO} (meV)	E_{LO} (meV)	Report
BDA	Pb	35.9	413.3	43	Ref. 15 (10.1021/acsnano.5c00144)
3AMP	Pb	47.3	208.7	33.3	Ref. 15 (10.1021/acsnano.5c00144)
3AMPy	Pb	33.6	134.8	24.5	Ref. 15 (10.1021/acsnano.5c00144)
DGA	Pb	31.4	58.1	Not reported	Ref. 16 (10.1002/adma.202208875)
DGA	Sn	224	1367	43.1	Reported here
3AMP	Sn	87.7	521	29.4	Fitting here using data from Ref. 17 (10.1021/acsnano.3c11125)
BDA	Sn	60.39	544.85	56.81	Ref. 18 (10.1039/d3ta00084b)
4F-PDMA	Sn	39.49	303.2	49.01	Reported here

References cited in the Supporting Information:

(1) Sheldrick, G. M. SHELXT – Integrated Space-Group and Crystal-Structure Determination. *Acta Cryst A* **2015**, *71* (1), 3–8. https://doi.org/10.1107/S2053273314026370.

(2) Sheldrick, G. M. Crystal Structure Refinement with SHELXL. *Acta Cryst C* **2015**, *71* (1), 3–8. https://doi.org/10.1107/S2053229614024218.

(3) Koegel, A. A.; Mozur, E. M.; Oswald, I. W. H.; Jalarvo, N. H.; Prisk, T. R.; Tyagi, M.; Neilson, J. R. Correlating Broadband Photoluminescence with Structural Dynamics in Layered Hybrid Halide Perovskites. *J. Am. Chem. Soc.* **2022**, *144* (3), 1313–1322. https://doi.org/10.1021/jacs.1c11217.

(4) Li, X.; Tao, Y.; Jiang, X.; Cai, G.; Gu, J.; Zheng, N.; Guan, Y.; Zhang, W.; Li, X.; Su, J.; Liu, Z.; Bian, Z.; Sun, J.; Li, C.; Fu, Y. Optical Bandgap Anomaly with Tuning Dimensionality in Germanium Perovskites: Interplay between Quantum Confinement and Lone Pair Expression. *Chem* **2024**, *10* (3), 891–909. https://doi.org/10.1016/j.chempr.2023.11.011.

(5) Kubelka, P.; Munk, F. Ein Beitrag Zur Optik Der Farbanstriche. *Z. Techn. Physik* **1931**, *12*, 593–601.

(6) Kresse, G.; Furthmüller, J. Efficient Iterative Schemes for Ab Initio Total-Energy Calculations Using a Plane-Wave Basis Set. *Phys. Rev. B* **1996**, *54* (16), 11169–11186. https://doi.org/10.1103/PhysRevB.54.11169.

(7) Blöchl, P. E. Projector Augmented-Wave Method. *Phys. Rev. B* **1994**, *50* (24), 17953–17979. https://doi.org/10.1103/PhysRevB.50.17953.

(8) Perdew, J. P.; Burke, K.; Ernzerhof, M. Generalized Gradient Approximation Made Simple. *Phys. Rev. Lett.* **1996**, *77* (18), 3865–3868. https://doi.org/10.1103/PhysRevLett.77.3865.

(9) Grimme, S.; Antony, J.; Ehrlich, S.; Krieg, H. A Consistent and Accurate *Ab Initio* Parametrization of Density Functional Dispersion Correction (DFT-D) for the 94 Elements H-Pu. *The Journal of Chemical Physics* **2010**, *132* (15), 154104. https://doi.org/10.1063/1.3382344.

(10) Gavnholt, J.; Olsen, T.; Engelund, M.; Schiøtz, J. *Δ* Self-Consistent Field Method to Obtain Potential Energy Surfaces of Excited Molecules on Surfaces. *Phys. Rev. B* **2008**, *78* (7), 075441. https://doi.org/10.1103/PhysRevB.78.075441.

(11) Perdew, J. P.; Ernzerhof, M.; Burke, K. Rationale for Mixing Exact Exchange with Density Functional Approximations. *J. Chem. Phys.* **1996**, *105* (22), 9982–9985. https://doi.org/10.1063/1.472933.

(12) Alkauskas, A.; Yan, Q.; Van de Walle, C. G. First-Principles Theory of Nonradiative Carrier Capture via Multiphonon Emission. *Phys. Rev. B* **2014**, *90* (7), 075202. https://doi.org/10.1103/PhysRevB.90.075202.

(13) Ouyang, R.; Curtarolo, S.; Ahmetcik, E.; Scheffler, M.; Ghiringhelli, L. M. SISSO: A Compressed-Sensing Method for Identifying the Best Low-Dimensional Descriptor in an Immensity of Offered Candidates. *Phys. Rev. Mater.* **2018**, *2* (8), 083802. https://doi.org/10.1103/PhysRevMaterials.2.083802.

(14) Purcell, T. A. r; Scheffler, M.; Carbogno, C.; Ghiringhelli, L. M. SISSO++: A C++ Implementation of the Sure-Independence Screening and Sparsifying Operator Approach. *Journal of Open Source Software* **2022**, *7* (71), 3960. https://doi.org/10.21105/joss.03960.

(15) Zhao, R.; Zhang, T.; Guo, L.; Wang, S.; Zhang, Y.; Li, P.; Song, Y. Manipulating Exciton–Phonon Coupling to Optimize Carrier Properties for High-Performance 2D Perovskite

Photodetectors. *ACS Nano* **2025**, *19* (12), 12182–12193. https://doi.org/10.1021/acsnano.5c00144.

(16) Zhang, B.; Zheng, T.; You, J.; Ma, C.; Liu, Y.; Zhang, L.; Xi, J.; Dong, G.; Liu, M.; Liu, S. (Frank). Electron-Phonon Coupling Suppression by Enhanced Lattice Rigidity in 2D Perovskite Single Crystals for High-Performance X-Ray Detection. *Adv. Mater.* **2023**, *35* (7), 2208875. https://doi.org/10.1002/adma.202208875.

(17) Liao, C.; Bernardi, S.; Bailey, C. G.; Chao, I. H.; Chien, S.-Y.; Wang, G.; Sun, Y.-H.; Tang, S.; Zheng, J.; Yi, J.; Yu, M.-H.; Russo, S. P.; Yen, H.-W.; McCamey, D. R.; Kennedy, B. J.; Widmer-Cooper, A.; Chueh, C.-C.; Ho-Baillie, A. W. Y. Piperidine and Pyridine Series Lead-Free Dion–Jacobson Phase Tin Perovskite Single Crystals and Their Applications for Field-Effect Transistors. *ACS Nano* **2024**, *18* (22), 14176–14186. https://doi.org/10.1021/acsnano.3c11125.

(18) Ji, H.; Liu, X.; Li, L.; Zhang, F.; Qin, L.; Lou, Z.; Li, D.; Hu, Y.; Hou, Y.; Teng, F. Two-Dimensional Layered Dion–Jacobson Phase Organic–Inorganic Tin Iodide Perovskite Field-Effect Transistors. *J. Mater. Chem. A* **2023**, *11* (14), 7767–7779. https://doi.org/10.1039/D3TA00084B.
